# Supplementary material for: Enhancing multiphoton upconversion through interfacial energy transfer in multilayered nanoparticles
Source: Nat Commun. 2020 Mar 4;11:1174. doi: 10.1038/s41467-020-14879-9 (PMC7055352; doi:10.1038/s41467-020-14879-9)
Supplement: Supplementary file 1 — Supplementary Information [file 41467_2020_14879_MOESM1_ESM.pdf]

SUPPLEMENTARY INFORMATION

Enhancing multiphoton upconversion through interfacial energy transfer in multilayered nanoparticles

Zhou et al.

[1]State Key Laboratory of Material Processing and Die & Mould Technology, School of Materials Science and Engineering, Huazhong University of Science and Technology, Wuhan 430074, China. [2]Beijing National Laboratory for Molecular Science, Key Laboratory of Photochemistry, Institute of Chemistry, Chinese Academy of Sciences, Beijing, 100190, China. [3]Key Laboratory for Biomedical Effects of Nanomaterials and Nanosafety, Institute of High Energy Physics and National Center for Nanosciences and Technology, Chinese Academy of Sciences, Beijing 100049, China. *e-mail: yingma@hust.edu.cn

Supplementary Methods

Materials. Lanthanide oxides (Ln: Y, Yb, Tm, Er, Tb), oleic acid (OA; 90%), oleylamine (OM; >80%) and 1-octadecene (ODE; >90%) were purchased from Sigma-Aldrich. CF_3COONa (NaTFA) and CF_3COOLi (LiTFA) were purchased from Alfa Aesar. Trifluoroacetic acid (99.5%) was purchased from Aladdin Inc. Ethanol, cyclohexane and Tween 20 (⩾99.5%) were purchased from Sinopharm Chemical Reagent Co., China. Hematoporphyrin monomethyl ether (HMME; ⩾95%) was purchased from Shanghai Shifeng Biological Technology Co., Ltd. Singlet oxygen sensor green reagent (SOSG) was purchased from Dalian Meilun Biotechnology Co., Ltd. All chemicals were used without further purification. Lanthanide trifluoroacetate (LnTFA) were prepared by the method we reported previously [(1)].

General procedure for synthesis of the core nanoparticles:

α-$NaYF_4$ UCNPs were chosen here due to their facile synthesis and controllability. The core nanoparticles were synthesized following a procedure reported by Yan et al.[(2)] with a slight modification. In a typical procedure to synthesize 11.1 nm-sized α-$NaYF_4$: 10 mol% Er nanoparticles, an aqueous solution containing 1 mmol NaTFA, 0.9 mmol YTFA and 0.1 mmol ErTFA was mixed with 5 mL OA, 5 mL OM and 10 mL ODE in a 50 mL flask. Then the mixture was heated at 130 ℃ for 30 min to form an optically transparent solution. Subsequently, the solution was heated to 290 °C and kept for 30 min under Ar flow with vigorous stirring before being cooled to room temperature. The resulting nanoparticles were precipitated by addition of ethanol, collected by centrifugation at 10000 rpm for 10 min, washed twice with a mixture of cyclohexane/ethanol, and then re-dispersed in 10 mL cyclohexane for later use. The synthetic procedure for other core nanoparticles is identical to that for α-$NaYF_4$: 10mol%Er except for the use of different precursors.

β-$NaYF_4$: 10 mol% Er nanoparticles were synthesized from α-$NaYF_4$: 10 mol% Er nanoparticles (acquired through the same procedure as above except for Na/Re ratio was controlled at 2.5) by using the following procedures. NaTFA aqueous solution (2.0 mmol) and 1.0 mmol α-$NaYF_4$:10 mol% Er nanoparticles dispersed in cyclohexane were first added into a mixture of 10 mL OA and 10 mL ODE in a 50mL flask. Then, the mixture was heated at 100 °C for 20 min to remove cyclohexane. After being further heated at 130 °C for 30 min, the mixture was finally heated to 320 °C and maintained at this temperature for 45 min under Ar flow with vigorous stirring. The resulting nanoparticles were precipitated, washed and re-dispersed in

cyclohexane as described above. For better control of the growth of β-$NaYF_4$ nanoparticles, a procedure reported by Steven Chu et al.(3) was also adopted with a slight modification to synthesize β-$NaYF_4$: 10 mol% Er core. An aqueous solution containing 0.9 mmol YCl_3 $6H_2O$ and 0.1 mmol $ErCl_3$ $6H_2O$ was firstly mixed with 10 mL OA and 10 mL ODE in a 50 mL flask, and then heated at 150 ℃ for 30 min to form an optically transparent solution. Subsequently, the solution was cooled to room temperature, and 6 mmol NH_4F and 3 mmol sodium oleate were quickly added into the reaction. Thereafter, the solution was heated to 120 ℃ and kept for 30 min, and then heated to 300 °C and kept for 60 min under Ar flow with vigorous stirring before being cooled to room temperature. The resulting nanoparticles were precipitated by addition of ethanol, collected by centrifugation, washed twice with a mixture of cyclohexane/ethanol, and then re-dispersed in 10 mL cyclohexane for later use.

General procedure for the synthesis of core-shell nanoparticles:

The as-synthesized $NaYF_4$:10%Er core nanoparticles (3 mL cyclohexane dispersion) were used as seeds to induce a subsequently epitaxial growth of $NaYbF_4$ shell layer. Taking the growth of 8.3-nm-thick $NaYbF_4$ shell layer as an example, an aqueous solution of YbTFA (4 mmol) and NaTFA (4 mmol) was mixed with OA (8 mL) and ODE (12 mL) in a 50 mL flask. The resulting mixture was heated to 130 °C and kept for 30min to form yellowish lanthanide-oleate complexes, followed by being cooled to room temperature. Subsequently, the as-prepared $NaYF_4$:Er core nanoparticles were added into the flask and the mixture were heated at 100 °C for 20 min to evaporate cyclohexane. Thereafter, the temperature of the mixture was raised to 290 °C at 15 °C/min and held at 290 °C for 30 min under an argon atmosphere. The resulting nanoparticles were precipitated by addition of ethanol, collected by centrifugation at 10000 rpm for 10 min, washed twice with a mixture of cyclohexane/ethanol, and then re-dispersed in cyclohexane (3 mL) for outmost shell growth. The synthetic procedure for other core-shell nanoparticles is identical to that for α-$NaYF_4$:10mol%Er@$NaYbF_4$ except for the use of different precursors or temperatures (β-phase).

General procedure for the synthesis of core-shell-shell nanoparticles:

Core-shell-shell nanoparticles were synthesized using a procedure similar to the procedure for core-shell nanoparticles. The as-synthesized $NaYF_4$:10%Er@$NaYbF_4$ core-shell nanoparticles (3 mL cyclohexane dispersion) were used as seeds to induce a subsequently epitaxial growth of $NaYF_4$ layer. NaTFA and YTFA were used as the

precursors for epitaxial growth of $NaYF_4$ shell. The synthetic procedure for other core-shell-shell nanoparticles is identical to that for α-$NaYF_4$:10mol%Er @$NaYbF_4$@$NaYF_4$ nanoparticles except for the use of different precursors or temperatures (β- phase).

General procedure for the synthesis of core-shell-shell-shell nanoparticles:

The procedure for synthesizing core-shell-shell-shell nanoparticles is identical to that for synthesis of core-shell-shell nanoparticles except for the use of different precursors.

Details for synthesis of various nanoparticles are shown in Table S1 and S2.

Nanocrystal Characterization.

For making reliable comparison, all kinds of nanoparticles were prepared from 0.3 mmol core nanoparticles (Table S1 and S2) and dispersed in 3 mL of cyclohexane for measurement. Considering the inevitable loss during shell growth, the concentration of core-shell-shell and core-shell-shell-shell nanoparticles should be smaller than that of core-shell nanoparticles, while nearly the same concentration will be obtained for various core-shell-shell nanoparticles (NPs). A Nikon digital camera was used to take photographs of the nanoparticles, which were excited by 980-nm laser at 15.2 W cm^{-2}. Exposure time: 0.01s. The quantum yield of the upconversion emission was measured on the same spectrometer combined with an integration sphere. Before measurement, the above NPs dispersions were diluted to minimize scattering of the excitation light. An excitation power density of 4.5 W cm^{-2} was used for all the measurements except for power dependent QY measurements for C_Y: 10%Er@S_{Yb}@S_Y NPs (d_{SYb} = 8.3±0.7, d_{SY} = 2.3±0.1). The quantum yield (QY) was calculated by the following equation

$$QY = \frac{visible\ photons\ emitted}{980\ nm\ photons\ absorbed} = \frac{I_{em,sample} - I_{em,reference}}{I_{ex,reference} - I_{ex,sample}}$$

where $I_{em,sample}$ and $I_{em,reference}$ are the integrated emission intensity in the range of 350-700 nm from the sample and the reference (~0), respectively. $I_{ex,reference}$ and $I_{ex,sample}$ are the integrated intensities of the excitation light in the presence of the reference and the sample, respectively. In the test, the cyclohexane was used as the reference.

Correction for spectral sensitivity.

Considering the diverse corrected UCL spectra for Tm^{3+} doped upconversion nanoparticles have been reported in previous articles (4-6), all spectra herein were collected with the mode of “emission correction off” if not specified to emphasize the

variation in the short wavelength. For avoiding confusion, typical UCL spectra were also collected with the mode of “emission correction on” for comparison.

Tween 20 modification of UCNPs.

In a typical surface modification process, 500 μL of Tween 20 was added into a 100 mL flask containing 50 mg of the synthesized UCNPs and 25 mL of cyclohexane under 10 min sonication. The milk-like dispersion was added into 50 mL of deionized water in a 100 mL flask for another 30 min sonication. After that, the flask was transferred into a 75 °C-water bath under stirring to evaporate the cyclohexane. After centrifugation, the UCNPs were washed with deionized water for two times. Tween 20-UCNPs were obtained via vacuum freeze-drying method.

Hematoporphyrin monomethyl ether (HMME) loading on UCNPs.

Loading of HMME onto Tween 20-UCNPs was carried out by soaking 5 mg of Tween 20-UCNPs with 8 mL of a HMME aqueous solution (the concentration of HMME is 0.188, 0.325, 0.563, 0.750 mmol L^{-1}, respectively) within 10 mL vials and stirring overnight at room temperature in the dark. The formed scarlet composites (HMME-UCNPs) were centrifugalized at 11 000 rps for 10 min, and the supernatants were also carefully collected for the loading percentage test, the HMME-UCNPs were washed with deionized water 3 times and dried via vacuum freeze drying method. The loading capacities of HMME on Tween 20-UCNPs were determined by absorption spectra of the HMME aqueous solution before and after loading. The calibration curves for absorption vs. concentration of HMME are shown in Supplementary Fig. 32. The photosensitizer loading percentage can then be derived using the following formula:

Photosensitizer loading

$$= \frac{\text{Mass of photosensitizer incorporated into nanoparticles}}{\text{Mass of nanoparticles}} \times 100$$

Detection of $^1O_2$ production of HMME-UCNPs in solution under 980 nm laser irradiation.

In a typical experiment, 2 mg of above HMME-UCNPs were suspended in 2 mL of a SOSG aqueous solution (the concentration of SOSG is 10 μmol L^{-1}). The mixture was injected into a quartz cuvette placed on a magnetic stirring apparatus and the solution was irradiated with an 980 nm laser at 24 W cm^{-2} for 5 min time intervals beginning from time (t) = 0 to 30 min, with the fluorescence emission of SOSG (excited by 380 nm) being measured between intervals using FLS980.

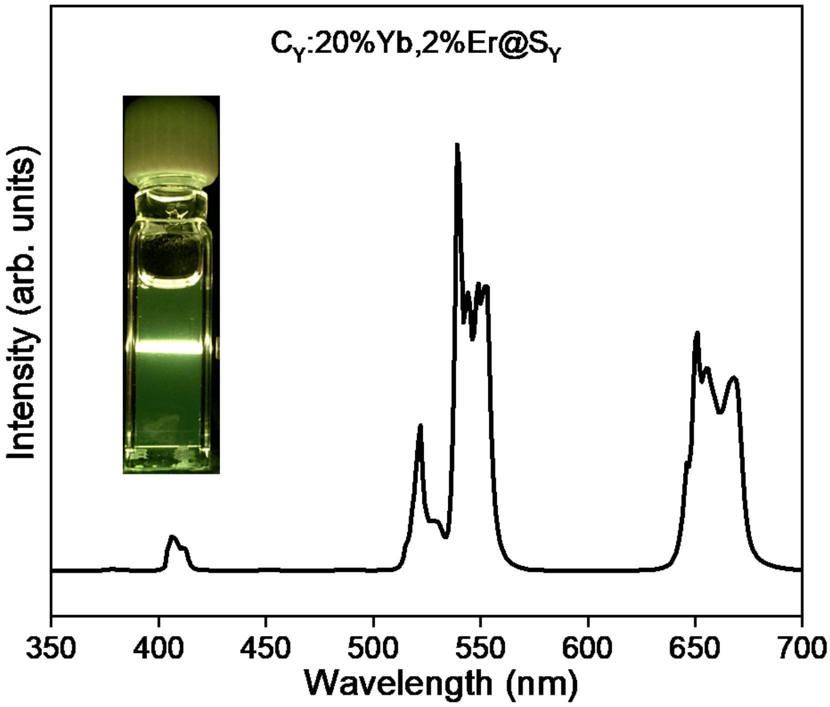

Supplementary Figure 1. Upconversion Emission of canonical core-shell nanoparticles. Emission spectrum and luminescent photograph for the $NaYF_4$:20% Yb, 2% Er@$NaYF_4$ core-shell nanoparticles.

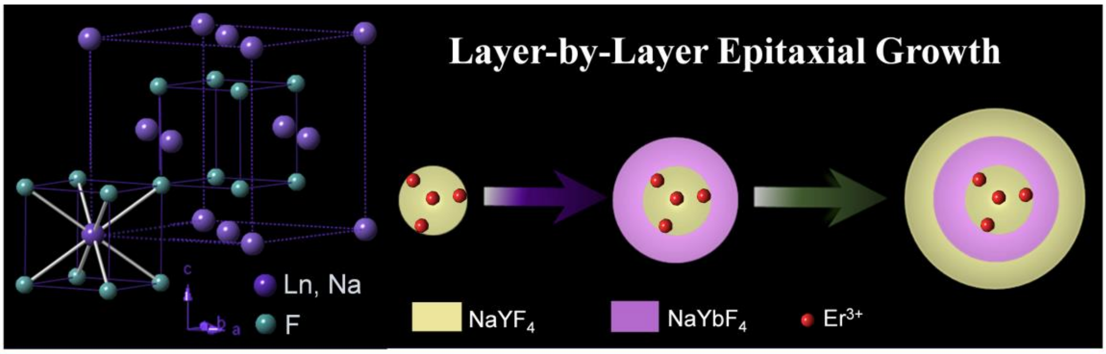

hkl	111	200	220	311	222	400
α-$NaYF_4$	3.16	2.74	1.93	1.65	1.58	1.37
α-$NaYbF_4$	3.13	2.71	1.91	1.63	1.56	1.35
Mismatch	1.01%	0.99%	1.0%	0.97%	1.01%	1.02%

Supplementary Figure 2. Epitaxial growth of core-shell-shell nanoparticles. Synthetic scheme for α-$NaYF_4$: Er@$NaYbF_4$@$NaYF_4$ core-shell-shell nanoparticles. The small lattice mismatches between α-$NaYF_4$ and α-$NaYbF_4$ crystals along different directions allow a layer-by-layer epitaxial growth of α-$NaYbF_4$ and α-$NaYF_4$ shells on pre-synthesized α-$NaYF_4$:10%Er core nanoparticles.

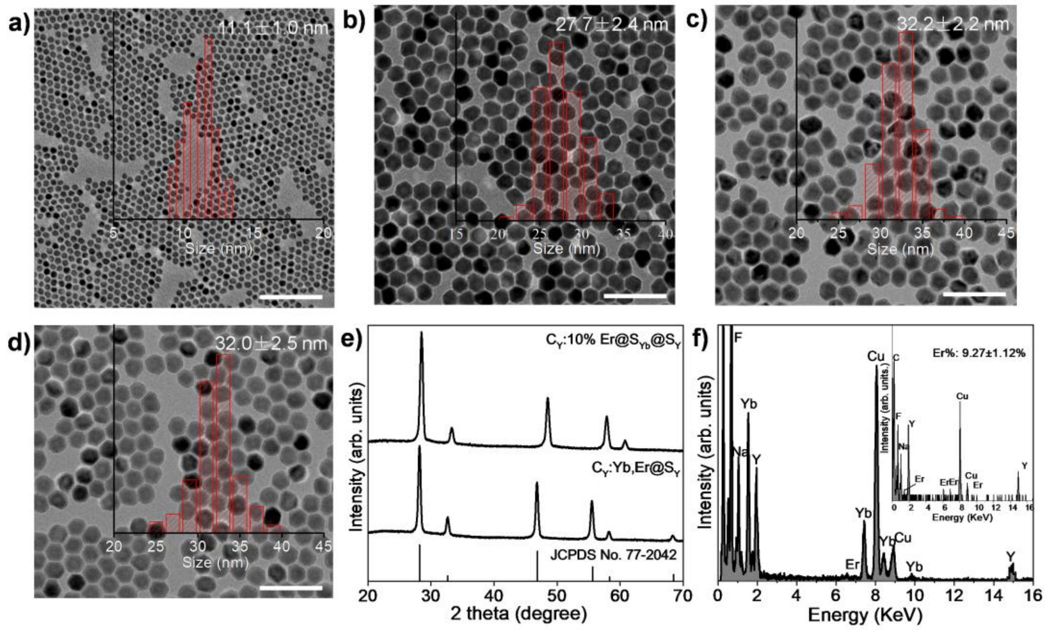

Supplementary Figure 3. Characterization of the core-shell-shell nanocrystals. TEM images and corresponding size distributions of $NaYF_4$:Er core (a), $NaYF_4$:Er@$NaYbF_4$ core-shell (b) and $NaYF_4$:Er@$NaYbF_4$@$NaYF_4$ core-shell-shell (c) and $NaYF_4$:Yb,Er@$NaYF_4$ core-shell (d) nanoparticles. The scale bars are 100 nm. (e) XRD patterns for the as-synthesized $NaYF_4$:Er@$NaYbF_4$@$NaYF_4$ and $NaYF_4$:Yb,Er@$NaYF_4$ nanoparticles. C_Y, C_{Yb}, S_{Yb} and S_Y are used to designate $NaYF_4$ core, $NaYbF_4$ core, $NaYbF_4$ shell and $NaYF_4$ shell, respectively. (f) The energy-dispersive X-ray spectra for the $NaYF_4$:Er@$NaYbF_4$@$NaYF_4$ and the $NaYF_4$:Er (inset) nanoparticles. The Er concentration of 9.27±1.12% determined from EDX analysis is comparable to the nominal concentration of 10%.

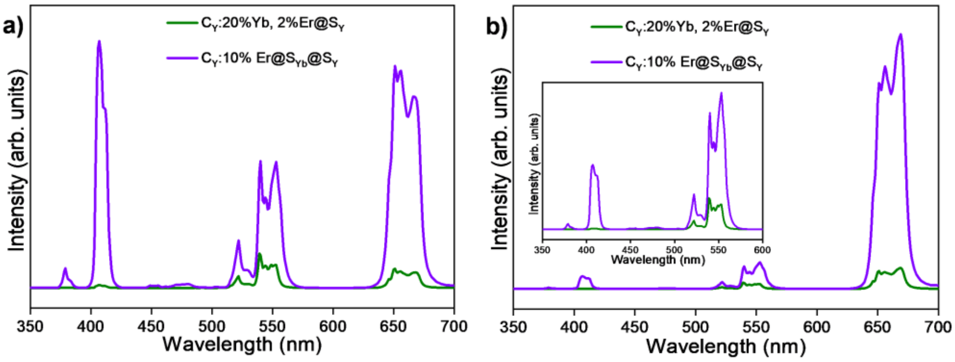

Supplementary Figure 4. Emission spectra collected in two modes. Comparison of upconversion emission spectra for $NaYF_4$:20%Yb, 2%Er@$NaYF_4$ core-shell (d_{SY} = 10.5±0.8 nm) and $NaYF_4$:10%Er@$NaYbF_4$@$NaYF_4$ core-shell-shell (d_{SYb} = 8.3±0.7 nm, d_{SY} = 2.3±0.1 nm; d_{SYb} and d_{SY} herein are used to designate the thickness of the intermediate $NaYbF_4$ and the outmost $NaYF_4$ shells, respectively.) nanoparticles collected with the mode "emission correction off" (a) and "emission correction on" (b).

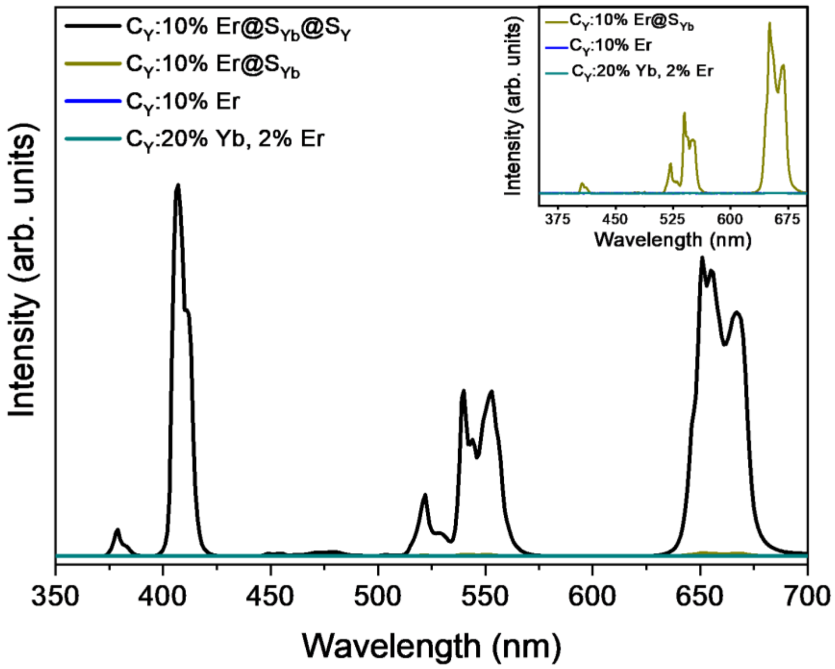

Supplementary Figure 5. Upconversion emission spectra for different structures. Upconversion emission spectra for $NaYF_4$:10%Er core (C_Y:10%Er), $NaYF_4$:10%Er@$NaYbF_4$ core-shell (C_Y:10%Er@S_{Yb}, d_{SYb} = 8.3±0.7 nm) and $NaYF_4$:10%Er@$NaYbF_4$@$NaYF_4$ core-shell-shell (C_Y:10%Er@S_{Yb}@S_Y, d_{SYb} = 8.3±0.7 nm, d_{SY} = 2.3±0.1 nm) nanoparticles. Upconversion emission spectrum for $NaYF_4$:Yb, Er core (C_Y:20%Yb, 2%Er) is also shown for comparison. Inset shows the enlarged spectra for some samples.

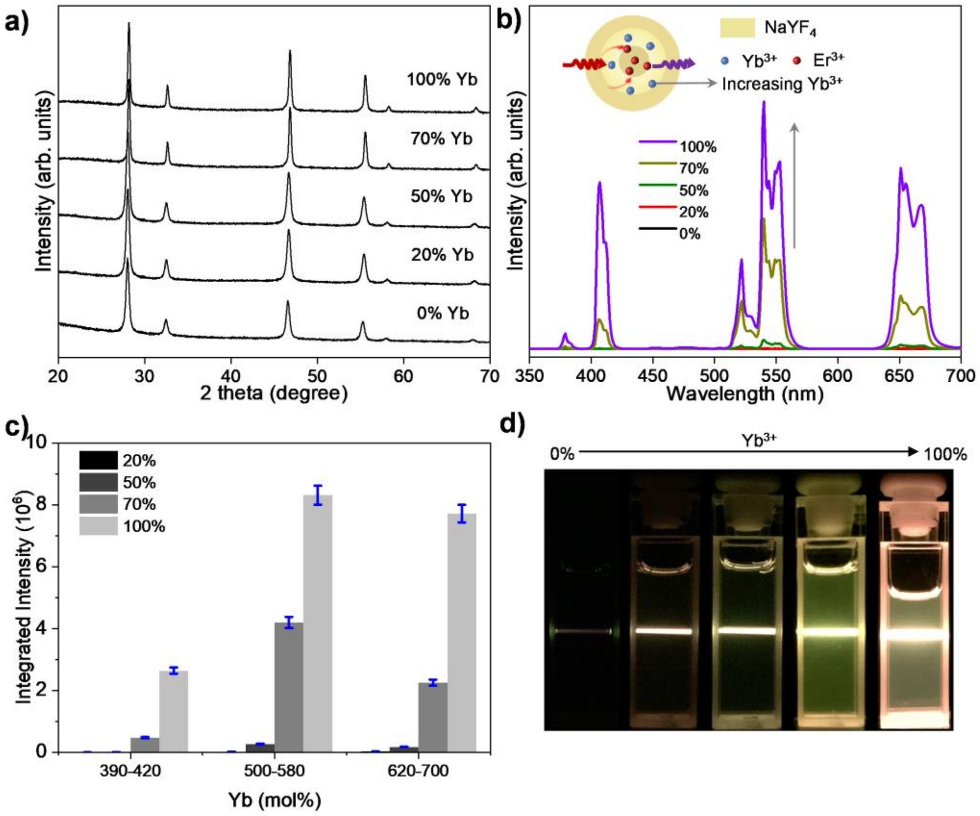

Supplementary Figure 6. Characterization of $NaYF_4$:10%Er@$NaYF_4$:Yb (0-100mol%) @$NaYF_4$ nanoparticles. The XRD patterns (a), upconversion emission spectra (b), emission peak intensity ratios (c) and luminescence photographs (d) for the $NaYF_4$:10%Er@$NaYF_4$:Yb (0-100mol%) @$NaYF_4$ core-shell-shell nanoparticles ($d_{SNaYF4:Yb}$ = 5.0 ±0.5 nm, d_{SY} = 2.4 ±0.2 nm) The $d_{SNaYF4:Yb}$ designates the thickness of the intermediate $NaYF_4$: Yb (0-100mol%).

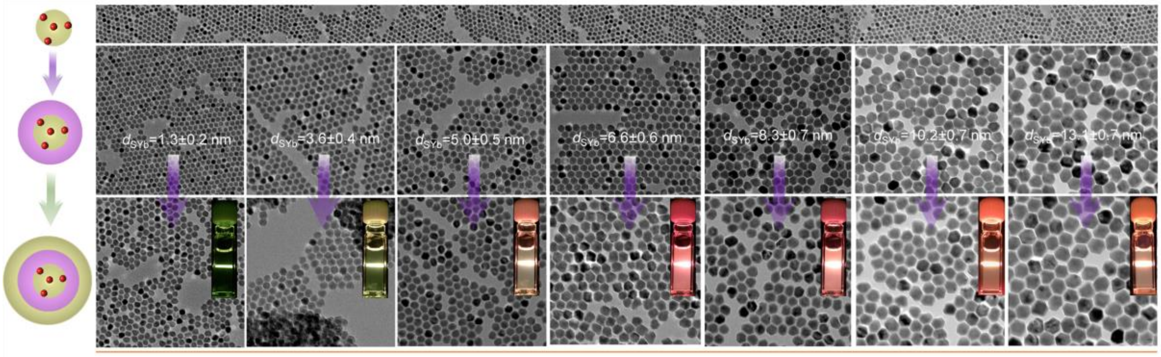

C	$C_Y@S_{Yb}$	Measured $\boldsymbol{d_{SYb}}$ (nm)	$C_Y@S_{Yb}@S_Y$	Measured $\boldsymbol{d_{SY}}$ (nm)
11.1±1.0	13.7±1.4	1.3±0.2	17.7±2.0	2.0±0.3
	18.3±1.8	3.6±0.4	23.1±2.4	2.4±0.3
	21.1±2.0	5.0±0.5	25.8±1.6	2.4±0.2
	24.3±2.3	6.6±0.6	29.8±2.1	2.7±0.1
	27.7±2.4	8.3±0.7	32.2±2.2	2.3±0.1
	31.5±2.4	10.2±0.7	36.1±2.3	2.3±0.1
	37.3±2.3	13.1±0.7	42.1±2.1	2.4±0.1

Supplementary Figure 7. Shell thickness determined by TEM observation. TEM images of the $NaYF_4$:10%Er@$NaYbF_4$@$NaYF_4$ core–shell–shell nanoparticles with varying $NaYbF_4$ shell thickness. The inert $NaYF_4$ shell thickness was controlled at 2.0~2.7 nm. The insets show that the emission firstly enhances and then decays with increasing $NaYbF_4$ shell thickness.

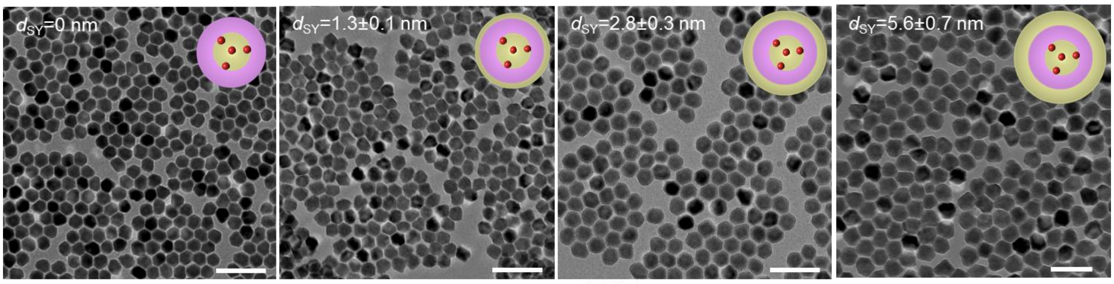

Supplementary Figure 8. Varying outmost $NaYF_4$ shell thickness. TEM images of the $NaYF_4$:10%Er@$NaYbF_4$@$NaYF_4$ core–shell–shell nanoparticles with varying $NaYF_4$ inert shell thickness. The $NaYbF_4$ shell thickness was controlled at ~ 8.3 nm. The scale bars are 100 nm.

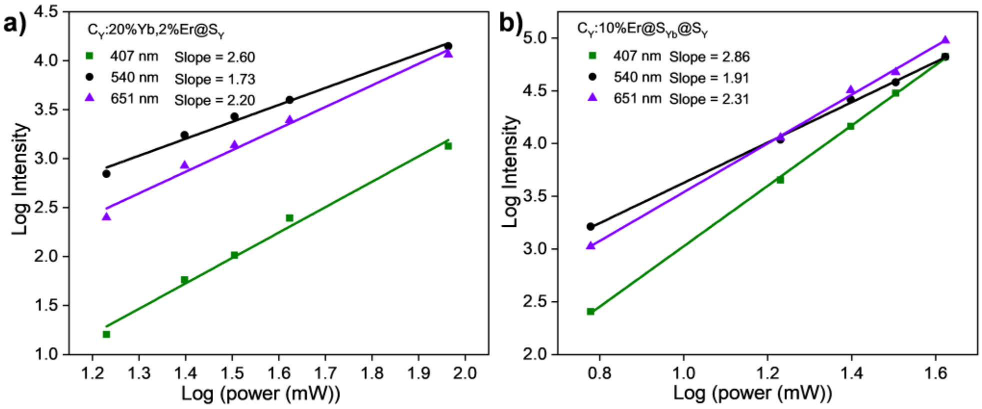

Supplementary Figure 9. Comparison of multiphoton upcoversion processes in two structures. Power-dependent upconversion emission intensity for the $^2H_{9/2}\rightarrow{}^4I_{15/2}$, $^4S_{3/2}\rightarrow{}^4I_{15/2}$ and $^4F_{9/2}\rightarrow{}^4I_{15/2}$ transitions of Er^{3+} from $NaYF_4$:Yb,Er@$NaYF_4$ (d_{SY} = 10.5±0.8 nm) (a) and $NaYF_4$:Er@$NaYbF_4$@$NaYF_4$ (d_{SYb} = 8.3±0.7 nm, d_{SY} = 2.3±0.1 nm) (b) nanoparticles.

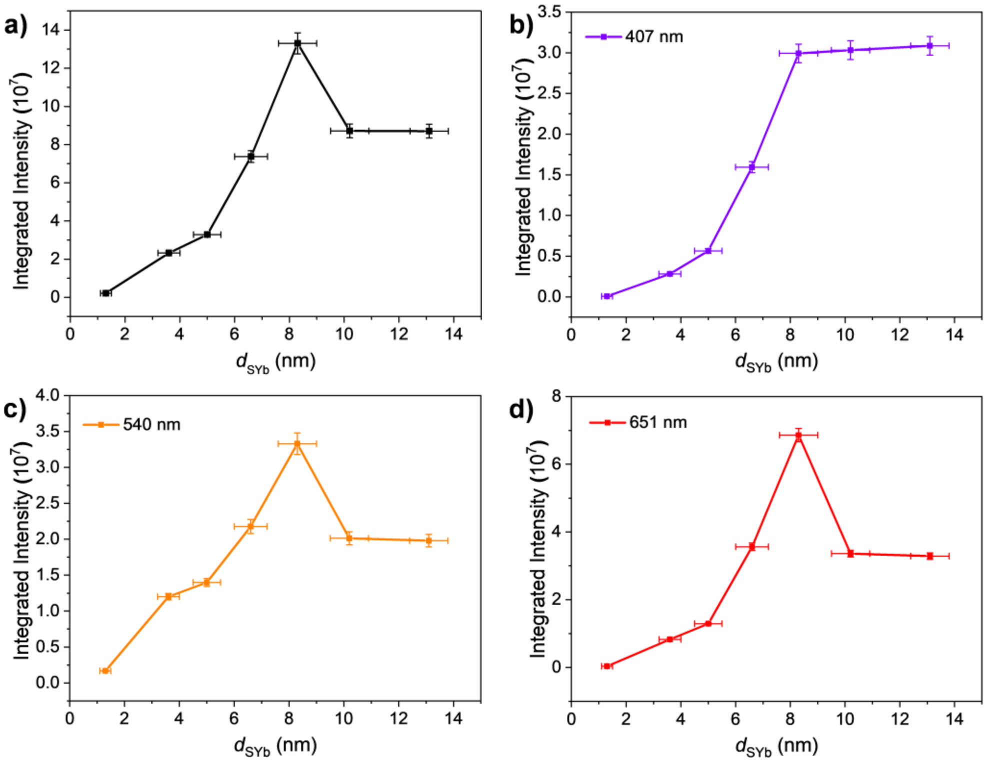

Supplementary Figure 10. Relationship of upconversion luminescence (UCL) intensity with sensitizer layer thickness. The d_{SYb}-dependent integrated intensity of UC emission for the $^2H_{9/2} \rightarrow {}^4I_{15/2}$, $^4S_{3/2} \rightarrow {}^4I_{15/2}$ and $^4F_{9/2} \rightarrow {}^4I_{15/2}$ transitions of Er^{3+} from $NaYF_4$:10%Er@$NaYbF_4$@$NaYF_4$ nanoparticles (d_{SY} = 2.0~2.7 nm).

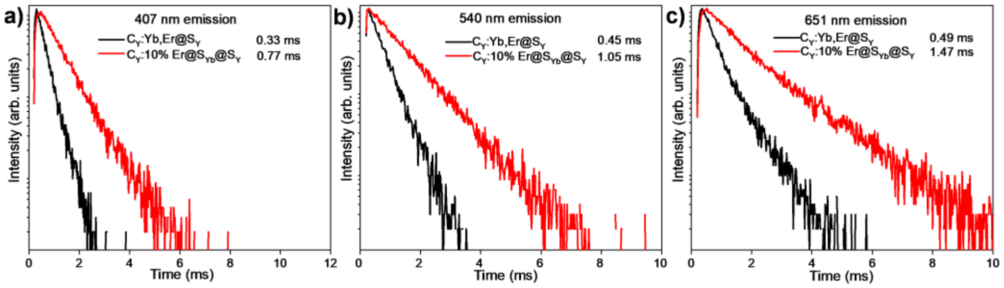

Supplementary Figure 11. Comparison of luminescence decay curves. Luminescence decay curves for $NaYF_4$:Er@$NaYbF_4$@$NaYF_4$ (d_{SYb} = 8.3±0.7 nm, d_{SY} = 2.3±0.1 nm) and $NaYF_4$:20%Yb, 2%Er@$NaYF_4$ (d_{SY} = 10.5±0.8 nm) nanoparticles. The decay curves were obtained by monitoring upconverted Er^{3+} emission at 407 nm (a), 540 nm (b) and 651 nm (c) under excitation of a pulsed laser at 980 nm, respectively.

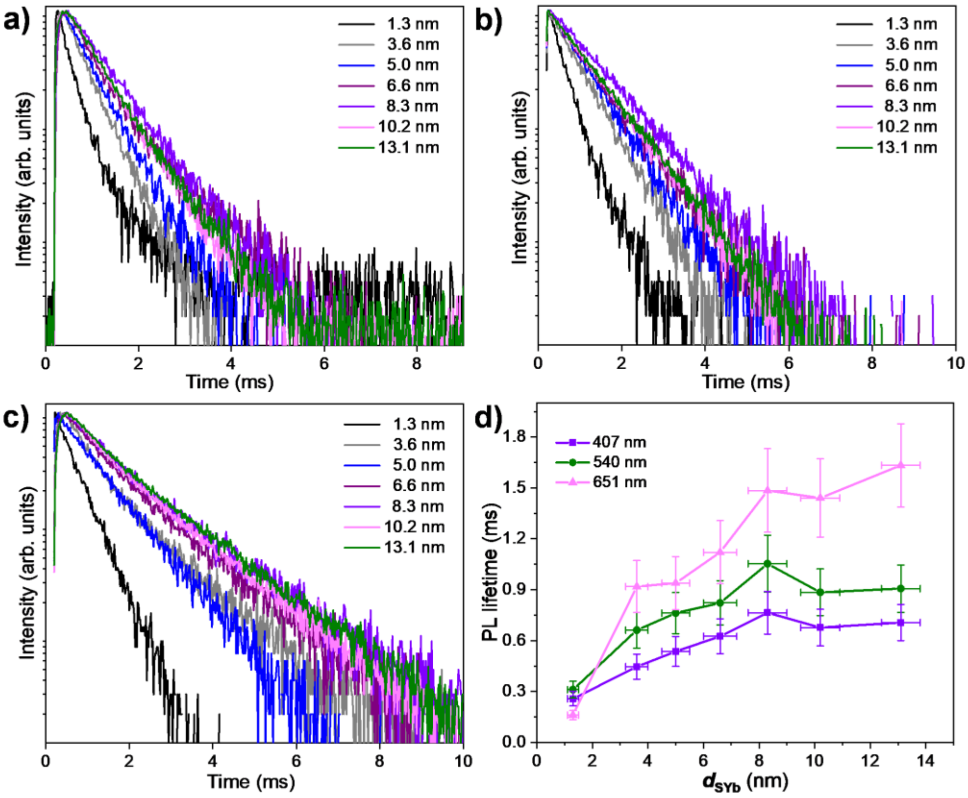

Supplementary Figure 12. Relationship of luminescence decay curves with sensitizer layer thickness. Luminescence decay curves for $NaYF_4$:10%Er@$NaYbF_4$@$NaYF_4$ nanoparticles with d_{SYb} varying from 1.3 to 13.1 nm (d_{SY} = 2.0~2.7 nm). The decay curves were obtained by monitoring upconverted Er^{3+} emission at 407nm (a), 540nm (b) and 651nm (c) under excitation of a pulsed laser at 980 nm, respectively. (d) PL lifetimes as a function of $NaYbF_4$ shell thickness.

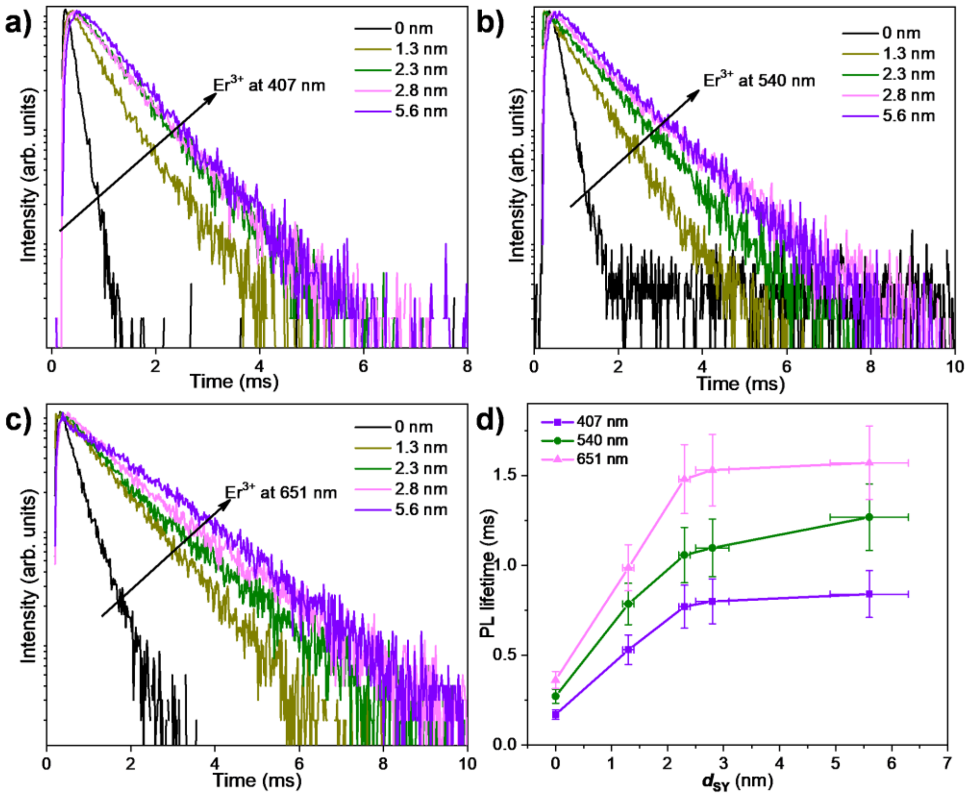

Supplementary Figure 13. Relationship of luminescence decay curves with outmost inert layer thickness. Luminescence decay curves for $NaYF_4$:10%Er@$NaYbF_4$ @$NaYF_4$ nanoparticles with d_{SY} varying from 0 to 5.6 nm (d_{SYb} = 8.3±0.7 nm). The decay curves were obtained by monitoring upconverted Er^{3+} emission at 407nm (a), 540nm (b) and 651nm (c) under excitation of a pulsed laser at 980 nm, respectively. (d) PL lifetimes as a function of $NaYF_4$ shell thickness.

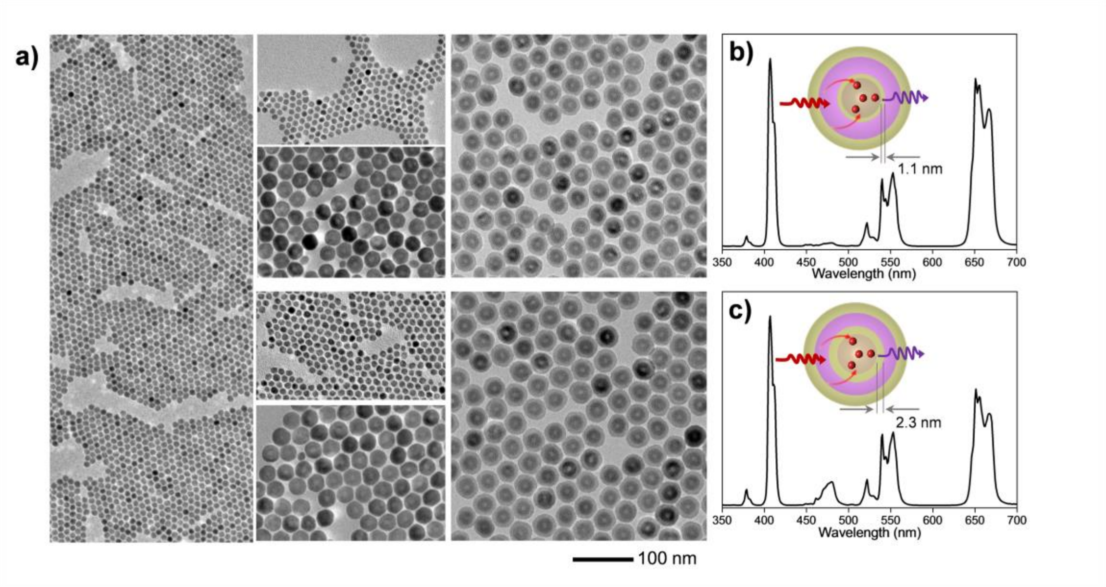

Supplementary Figure 14. UCL for multilayered structures with different intermediate inert layer thicknesses. (a) TEM images of the $NaYF_4$:10%Er@$NaYF_4$@$NaYbF_4$@$NaYF_4$ tetra-layered nanoparticles with 1.1 (top-right) and 2.3 nm-thick (bottom-right) $NaYF_4$ interlayer. TEM image of the cores is shown on the left. (b-c) Upconversion emission spectra indicate that insertion of another empty shell ($NaYF_4$ interlayer) has little effect on the population of different Er^{3+} energy levels but greatly reduces UCL intensity and quantum yield.

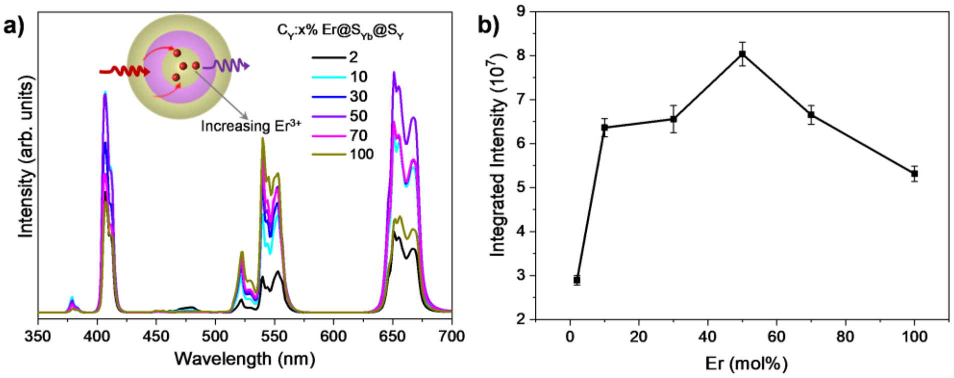

Supplementary Figure 15. Effect of Er^{3+} concentration on UCL intensity. (a) Upconversion emission spectra for the $NaYF_4$:Er (2-100mol%) @$NaYbF_4$@$NaYF_4$ core-shell-shell nanoparticles (d_{SYb} = 8.3±0.7 nm, d_{SY} = 2.3±0.1 nm). (b) The integrated emission intensities as a function of Er^{3+} concentration.

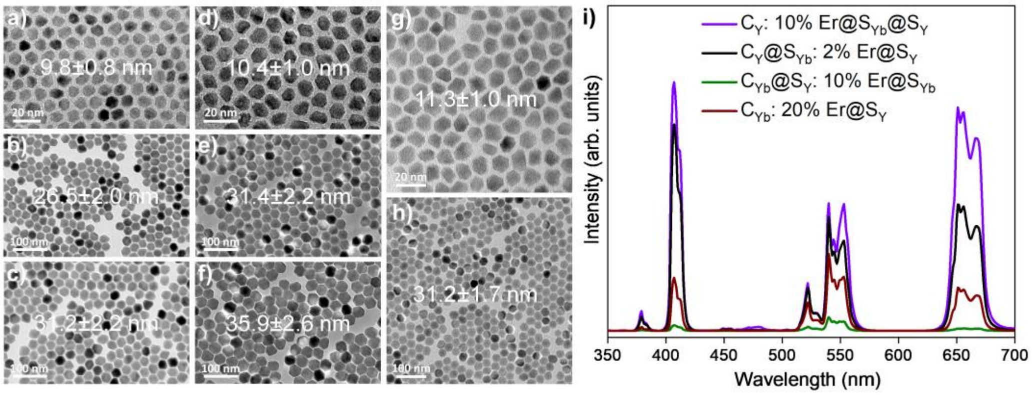

Supplementary Figure 16. A comparison of α-$NaYF_4$:10%Er@$NaYbF_4$@$NaYF_4$ nanoparticles and the recently reported new nanostructures.[3,9,10] (a-h) TEM images of α-$NaYF_4$@$NaYb_{0.98}Er_{0.02}F_4$@$NaYF_4$ (C_Y@S_{Yb}: 2%Er@S_Y) (a-c for the core, core-shell and core-shell-shell nanostructures), α-$NaYbF_4$@$NaY_{0.9}Er_{0.1}F_4$@$NaYbF_4$ (C_{Yb}@S_Y: 10%Er@S_{Yb}) (d-f for the core, core-shell and core-shell-shell nanostructures) and α-$NaYb_{0.8}Er_{0.2}F_4$@$NaYF_4$ (C_{Yb}: 20%Er@S_Y) (g, h for the core and core-shell nanostructures). (i) Upconversion emission spectra for these UCNPs upon 980-nm excitation (24.0 W cm^{-2}). The $NaYF_4$:10%Er@$NaYbF_4$@$NaYF_4$ nanoparticles emit the strongest UCL, demonstrating their superior photon upconversion efficiency over the others. It should be noted that α-$NaY_{0.8}Yb_{0.2}F_4$@$NaY_{0.98}Er_{0.02}F_4$@$NaY_{0.8}Yb_{0.2}F_4$ UCNPs might emit stronger UCL than C_{Yb}@S_Y: 10%Er@S_{Yb} UCNPs due to much weaker quenching effect. The same doping concentrations of Er (10%) and Yb (100%) were adopted here to validate this structure is not efficient for suppressing concentration quenching.

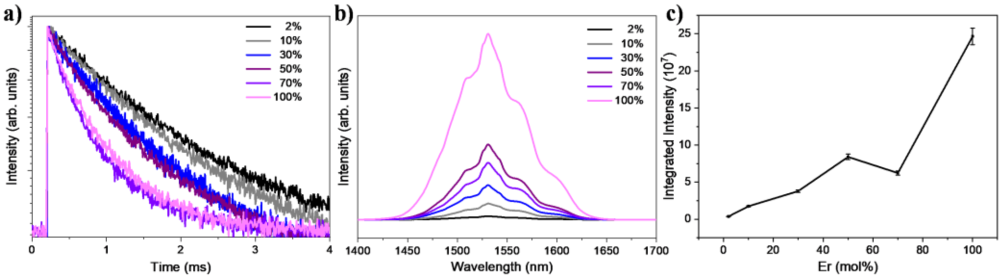

Supplementary Figure 17. Near Infrared (NIR) luminescence of core-shell-shell nanoparticles. (a-b) Luminescence decay curves of emission at 1000 nm (a) and NIR downshifting luminescence from Er^{3+} $^4I_{13/2}$ energy level for the $NaYF_4$:Er (2-100mol%) @$NaYbF_4$@$NaYF_4$ core-shell-shell nanoparticles (d_{SYb} = 8.3±0.7 nm, d_{SY} = 2.3±0.1 nm). (c) The integrated NIR emission intensities as a function of Er^{3+} concentration.

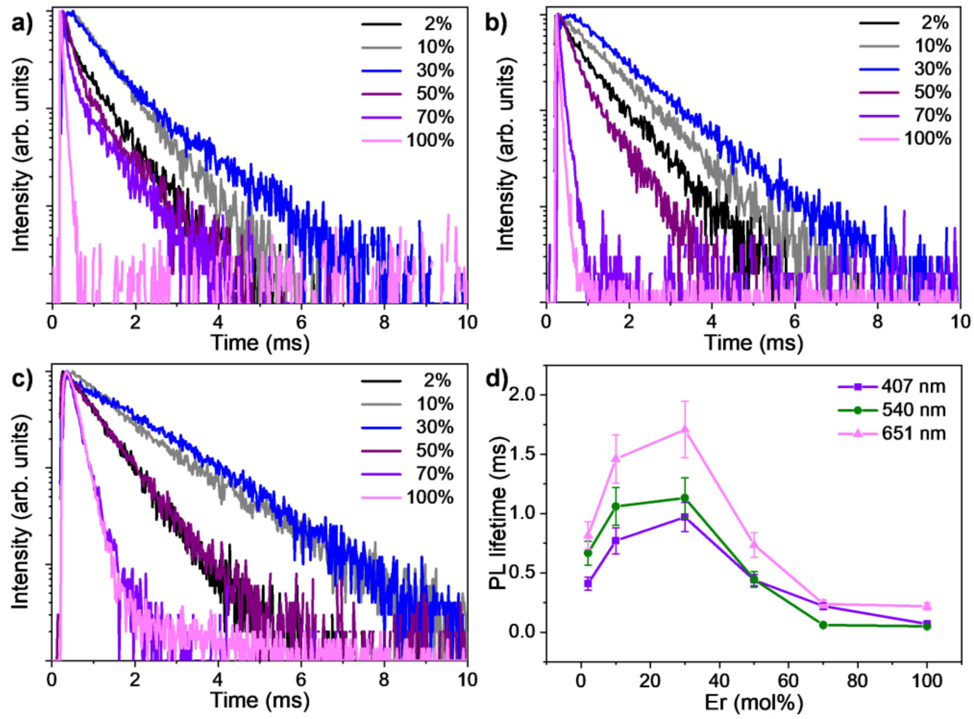

Supplementary Figure 18. Relationship of luminescence decay curves with Er^{3+} concentration. Luminescence decay curves of emission at 407 (a), 540 (b) and 651 nm (c) for the $NaYF_4$:Er (2-100mol%) @$NaYbF_4$@$NaYF_4$ core-shell-shell nanoparticles (d_{SYb} = 8.3±0.7 nm, d_{SY} = 2.3±0.1 nm). (d) PL lifetimes as a function of Er^{3+} concentration.

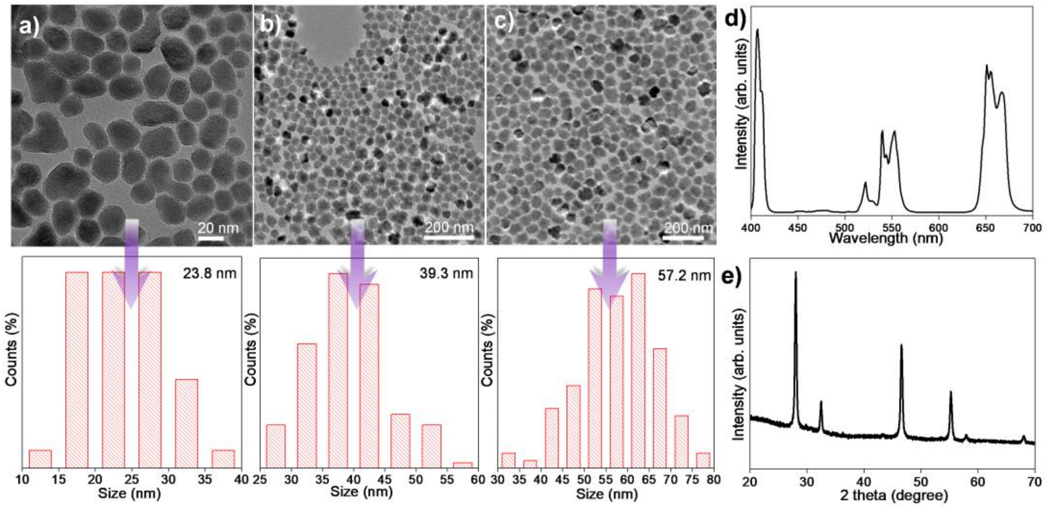

Supplementary Figure 19. UCL of larger sized core-shell-shell nanoparticles. Morphology and Photoluminescence spectrum of larger $NaYF_4$:10%Er@$NaYbF_4$@$NaYF_4$ core-shell-shell nanoparticles indicate the key roles of $NaYbF_4$ shell thickness in enhancing multiphoton emission. TEM images and their corresponding size distributions of the $NaYF_4$:10%Er (a), $NaYF_4$:10%Er@$NaYbF_4$ (b) and $NaYF_4$:10%Er@$NaYbF_4$@$NaYF_4$ (c) nanoparticles. The upconversion emission spectrum (d) and XRD pattern (e) for the $NaYF_4$:10%Er@$NaYbF_4$@$NaYF_4$ nanoparticles.

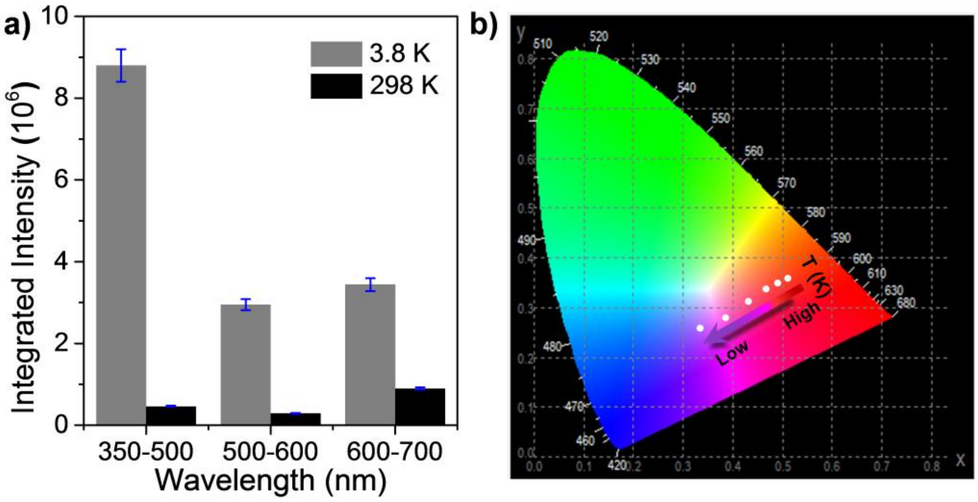

Supplementary Figure 20. Comparison of UCL at 3.8 and 298 K. (a) The integrated intensity of the emission bands determined from the photoluminescence spectra of α-$NaYF_4$:10%Er@$NaYbF_4$@$NaYF_4$ core-shell-shell nanoparticles (d_{SYb} = 8.3±0.7 nm, d_{SY} = 2.3±0.1 nm) at 3.8 and 298 K, respectively. (b) CIE chromaticity diagram shows the luminescent color of the $NaYF_4$: 10%Er@$NaYbF_4$@$NaYF_4$ nanoparticles at different temperatures.

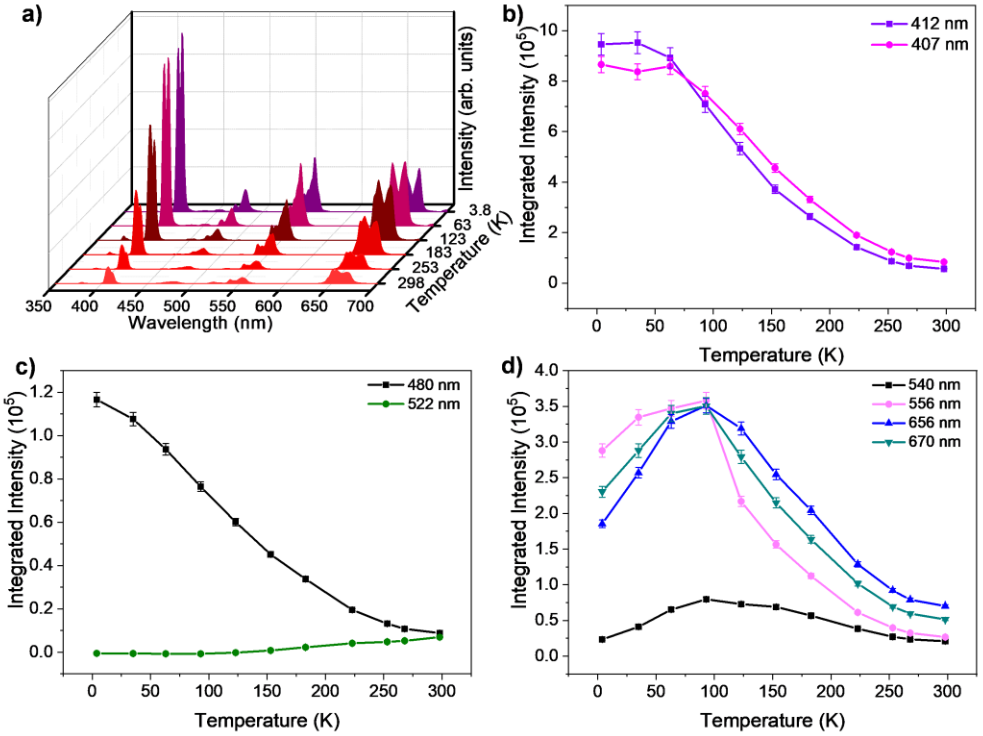

Supplementary Figure 21. Variation of UCL for core-shell-shell nanoparticles with decreasing temperature. (a) The representative emission spectra of α-$NaYF_4$: 10% Er@$NaYbF_4$@$NaYF_4$ core-shell-shell nanoparticles (d_{SYb} = 8.3±0.7 nm, d_{SY} = 2.3±0.1 nm) recorded from 3.8 to 298 K. (b-d) Temperature-dependent peak intensity of $^2H_{9/2}$, $^2F_{7/2}$, $^2H_{11/2}$, $^4S_{3/2}$ and $^4F_{9/2}$ to $^4I_{15/2}$ transitions for α-$NaYF_4$: 10%Er@$NaYbF_4$@$NaYF_4$ core-shell-shell nanoparticles.

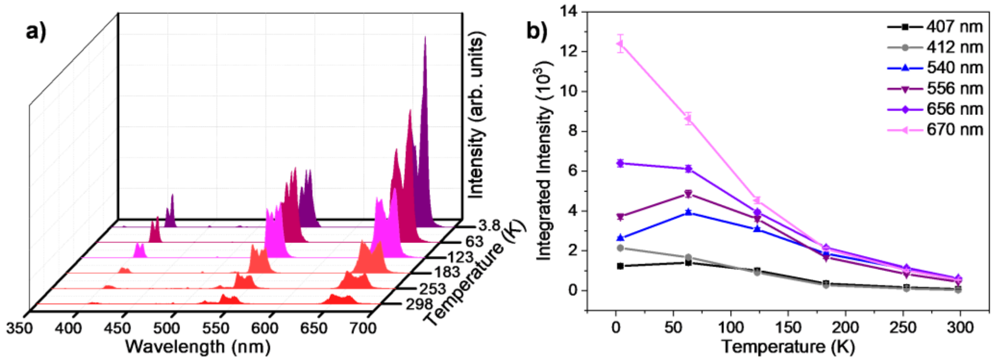

Supplementary Figure 22. Variation of UCL for core-shell nanoparticles with decreasing temperature. (a) The representative emission spectra of α-$NaYF_4$: 20% Yb, 2% Er@$NaYF_4$ core-shell nanoparticles (d_{SY} = 10.5 ±0.8 nm) recorded from 3.8 to 298 K. (b) Temperature-dependent peak intensity of $^2H_{9/2}$, $^2H_{11/2}$, $^4S_{3/2}$ and $^4F_{9/2}$ to $^4I_{15/2}$ transitions for α-$NaYF_4$: 20% Yb, 2% Er@$NaYF_4$ core-shell nanoparticles.

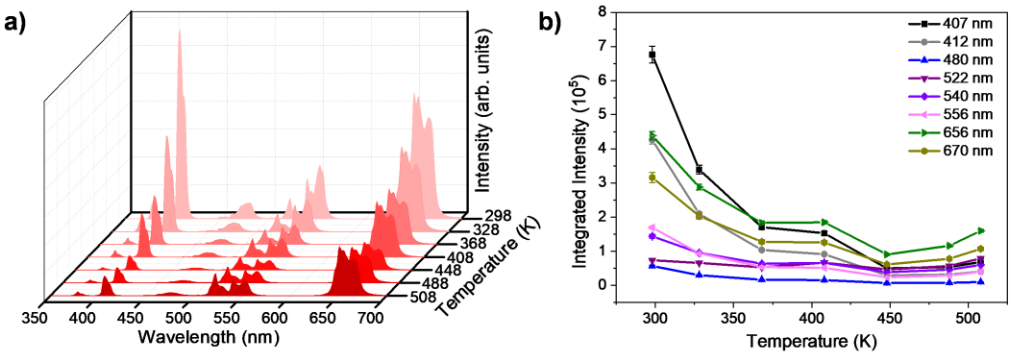

Supplementary Figure 23. Variation of UCL for core-shell-shell nanoparticles with increasing temperature. (a) The representative emission spectra of α-$NaYF_4$: 10% Er@$NaYbF_4$@$NaYF_4$ core-shell-shell nanoparticles (d_{SYb} = 8.3±0.7 nm, d_{SY} = 2.3±0.1 nm) recorded from 298 to 508 K. (b) Temperature-dependent peak intensity of $^2H_{9/2}$, $^2H_{11/2}$, $^4S_{3/2}$ and $^4F_{9/2}$ to $^4I_{15/2}$ transitions for α-$NaYF_4$: 10% Er@$NaYbF_4$@$NaYF_4$ core-shell-shell nanoparticles. The temperature dependent spectra demonstrate obvious thermal quenching effect. Abnormal intensity increase at temperatures higher than 488 K may be due to surface defects minimization, as reported previously (7).

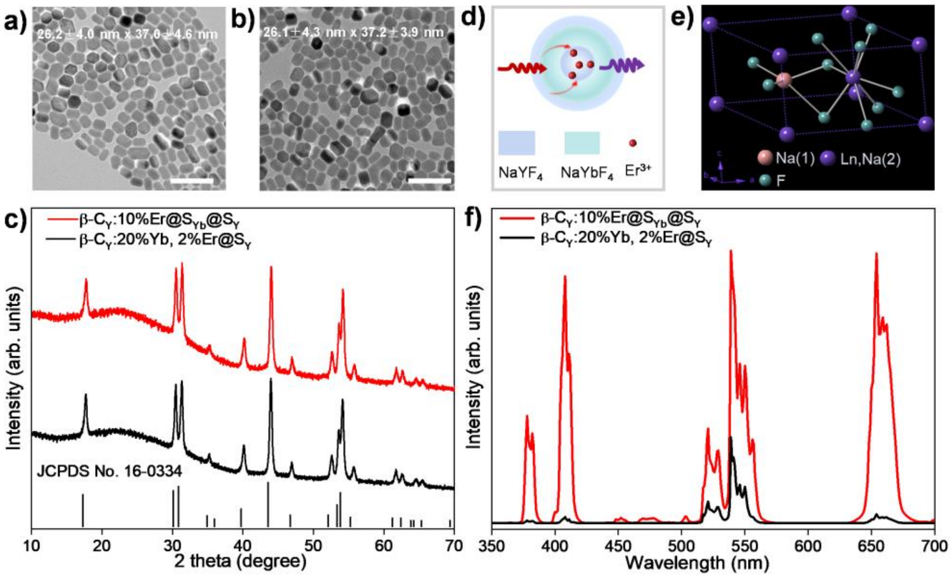

Supplementary Figure 24. Enhanced multiphoton upconversion emission of Er^{3+} in a β-$NaLnF_4$ host lattice. TEM images of (a) β-$NaYF_4$:Er@$NaYbF_4$@$NaYF_4$ and (b) β-$NaYF_4$:Yb,Er@$NaYF_4$ nanoparticles and (c) corresponding XRD patterns. The scale bars are 100 nm. (d) Schematic representation of the core-shell-shell structure for enhanced multiphoton upconversion in a β-$NaLnF_4$-based nanoparticle. (e) Schematic representation of the β-$NaYF_4$ crystal structure. (f) Upconversion emission spectra showing significantly enhanced red and violet emission from the as-synthesized β-$NaYF_4$:Er@$NaYbF_4$@$NaYF_4$ nanoparticles compared with that from β-$NaYF_4$:Yb,Er@$NaYF_4$ nanoparticles.

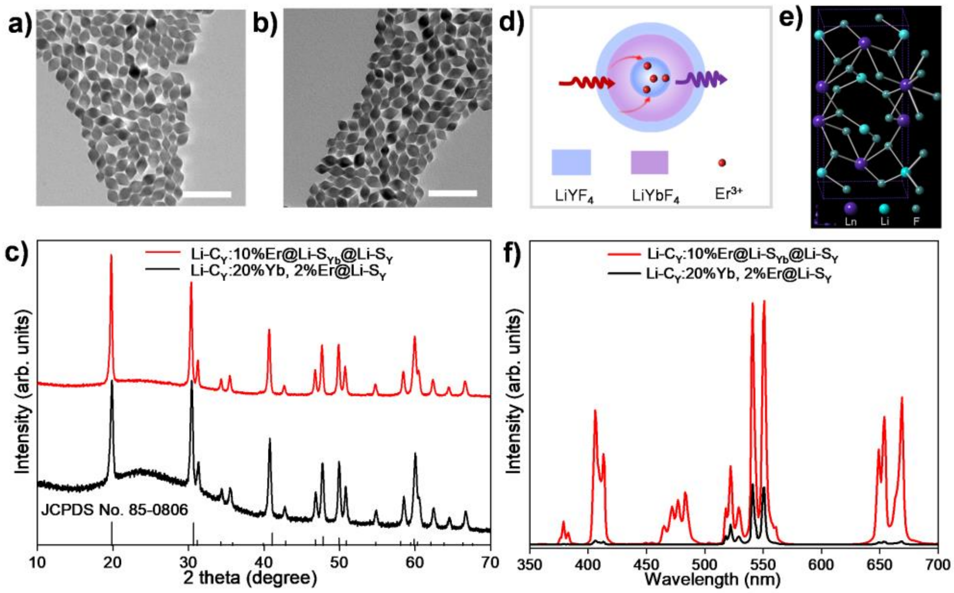

Supplementary Figure 25. Enhanced multiphoton upconversion emission of Er^{3+} in a $LiLnF_4$ host lattice. TEM images of (a) $LiYF_4$:Er@$LiYbF_4$@$LiYF_4$ and (b) $LiYF_4$:Yb,Er@$LiYF_4$ nanoparticles and (c) corresponding XRD patterns. The scale bars are 100 nm. (d) Schematic representation of the core-shell-shell structure for enhanced multiphoton upconversion in a $LiLnF_4$-based nanoparticle. (e) Schematic representation of the tetragonal-phase $LiYF_4$ crystal structure. (f) Upconversion emission spectra showing the as-synthesized $LiYF_4$:Er@$LiYbF_4$@$LiYF_4$ nanoparticles emit significantly enhanced red and violet emission compared with the $LiYF_4$:Yb,Er@$LiYF_4$ nanoparticles.

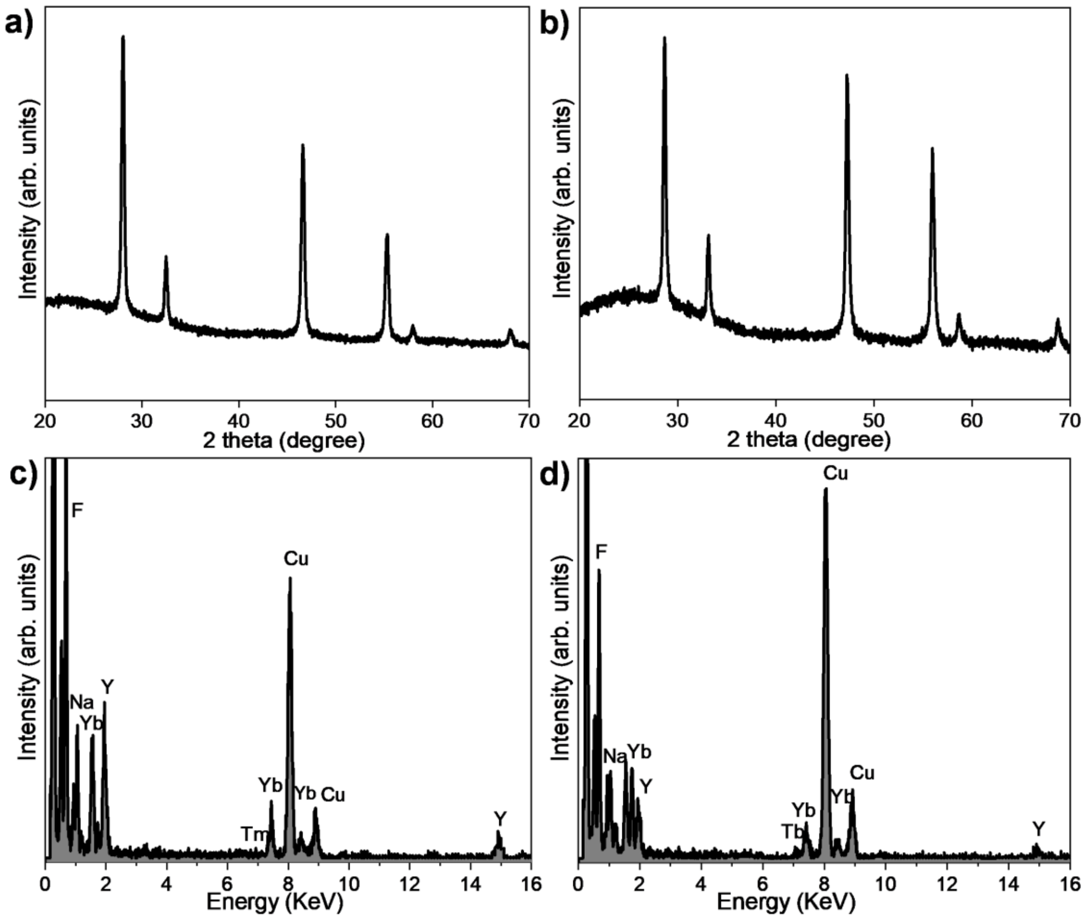

Supplementary Figure 26. Characterization of Tm^{3+} and Tb^{3+} doped nanocrystals. XRD and EDX patterns for α-$NaYF_4$: Tm@$NaYbF_4$@$NaYF_4$ (a, c) and α-$NaYF_4$: Tb@$NaYbF_4$@$NaYF_4$ (b, d) core-shell-shell nanoparticles.

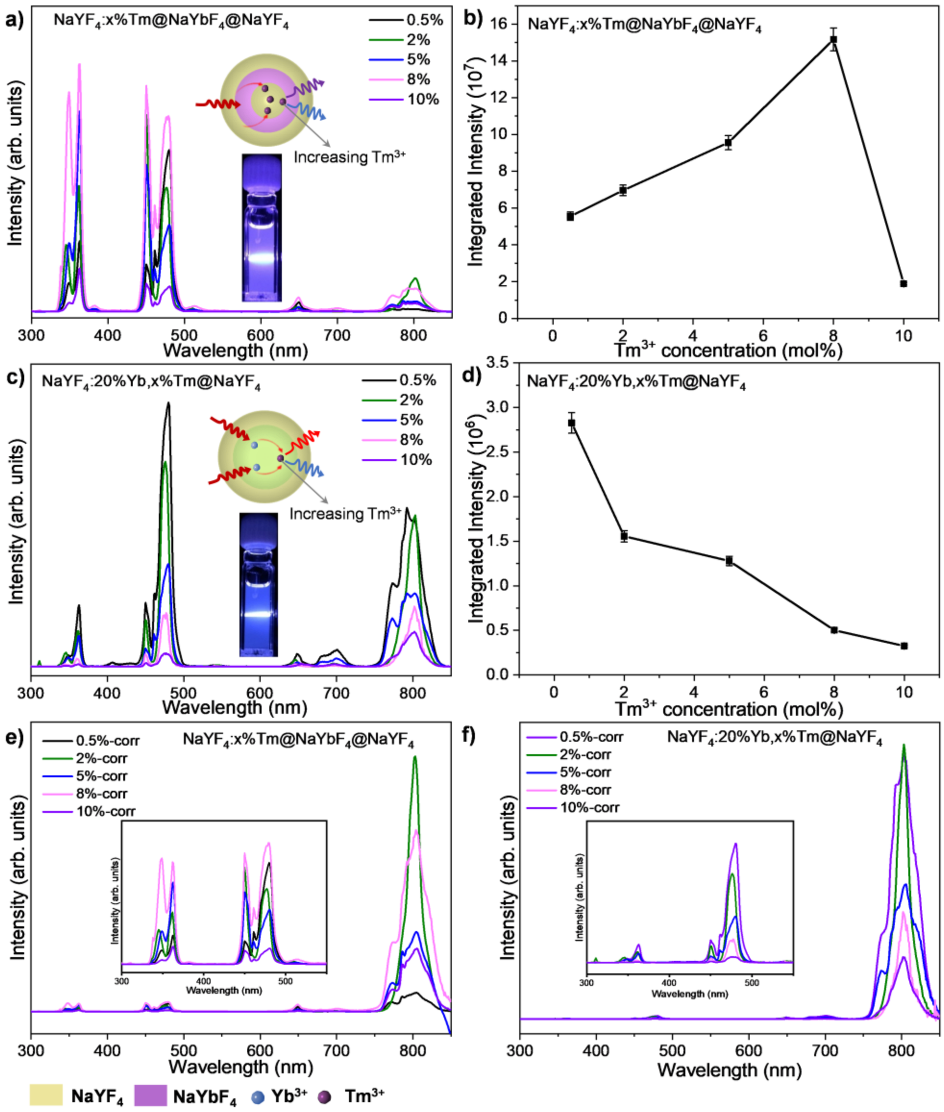

Supplementary Figure 27. Enhanced multiphoton upconversion in Tm^{3+}-doped core-shell-shell nanoparticles. Upconversion emission spectra and integrated intensity in the range of 300-600 nm for α-$NaYF_4$:Tm@$NaYbF_4$@$NaYF_4$ (d_{SYb} = 8.3±0.7 nm, d_{SY} = 2.3±0.1 nm) (a, b) and α-$NaYF_4$:Yb,Tm@$NaYF_4$ (d_{SY} = 10.5±0.8 nm) (c, d) nanoparticles at different Tm^{3+} doping concentrations showing suppression of luminescence quenching in α-$NaYF_4$:Tm@$NaYbF_4$@$NaYF_4$ nanoparticles at Tm^{3+} doping concentration below 8%. The inset: luminescence photographs of α-$NaYF_4$:8%Tm@$NaYbF_4$@$NaYF_4$ (a) and α-$NaYF_4$:Yb,0.5%Tm@$NaYF_4$ (c) nanoparticles. (e, f) The corresponding UCL spectra collected with the mode “emission correction on”.

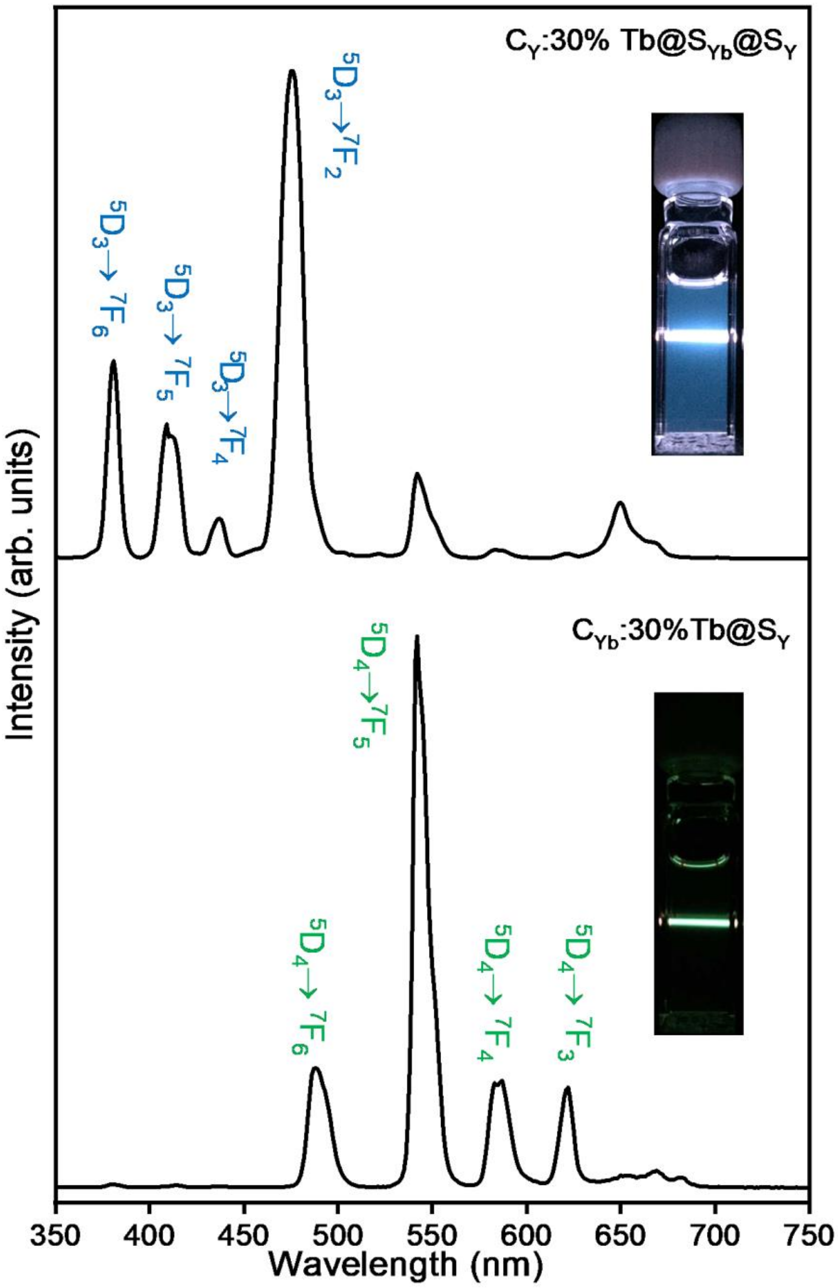

Supplementary Figure 28. Enhanced multiphoton upconversion in Tb^{3+}-doped core-shell-shell nanoparticles. Upconversion emission spectra for α-$NaYF_4$:Tb@$NaYbF_4$@$NaYF_4$ (top, d_{SYb} = 8.3±0.7 nm, d_{SY} = 2.3±0.1 nm) and α-$NaYbF_4$:Tb@$NaYF_4$ (bottom, d_{SY} = 10.5±0.8 nm) nanoparticles at a Tb^{3+} doping level of 30 mol%. The inset: luminescent photographs of the corresponding nanoparticles.

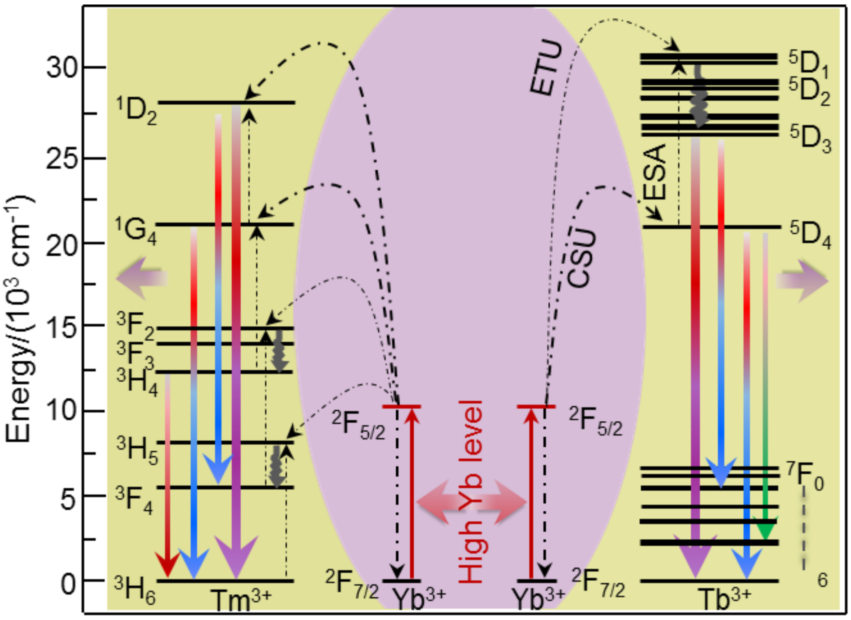

Supplementary Figure 29. UCL mechanism in core-shell-shell nanoparticles. Simplified energy level diagram shows enhanced multiphoton upconversion from Yb^{3+} to Tm^{3+}/Tb^{3+} in α-$NaYF_4$: Tm(Tb)@$NaYbF_4$@$NaYF_4$ core-shell-shell nanoparticles (d_{SYb} = 8.3±0.7 nm, d_{SY} = 2.3±0.1 nm).

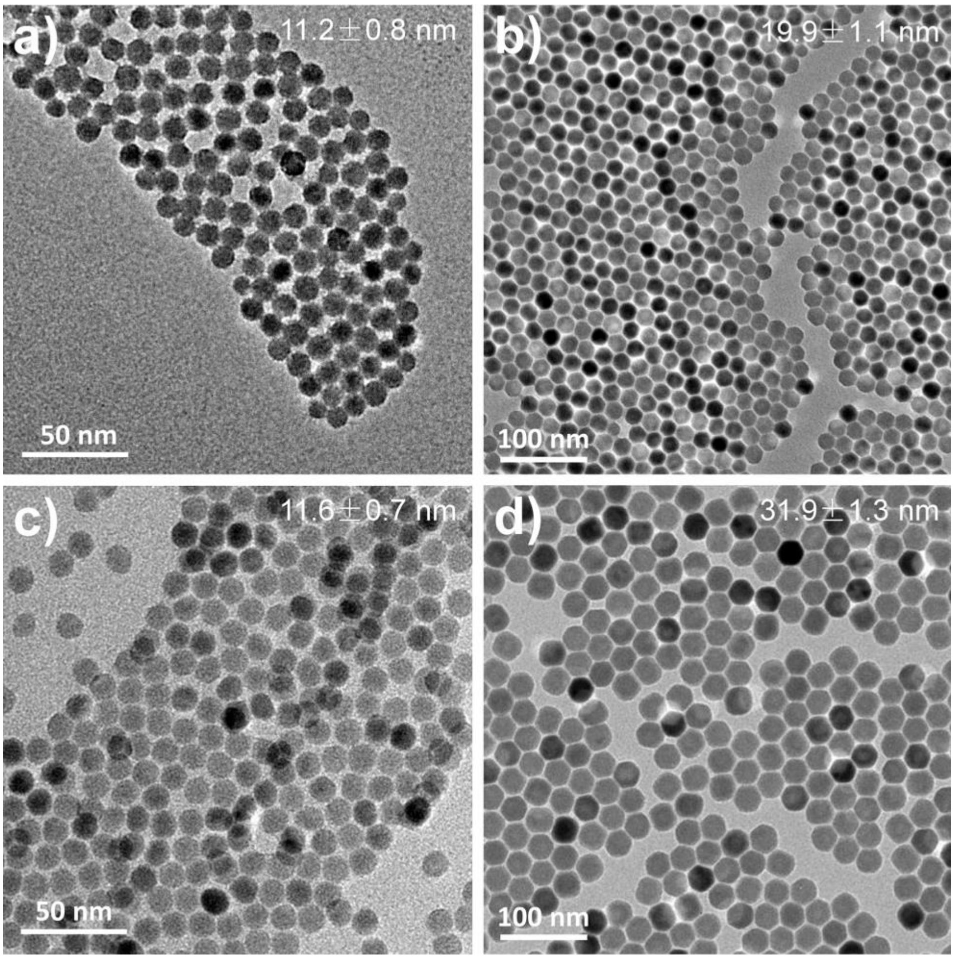

Supplementary Figure 30. Standard core-shell sample and β-phase core-shell-shell sample. TEM images of (a) β-$NaYF_4$:18%Yb, 2%Er, (b) β-$NaYF_4$:18%Yb, 2%Er@$NaYF_4$ (a standard sample synthesized according to ref 8), (c) β-$NaYF_4$:10%Er and (d) β-$NaYF_4$:10%Er@$NaYbF_4$@$NaYF_4$ nanoparticles (synthesized by a procedure similar to that reported in ref 3).

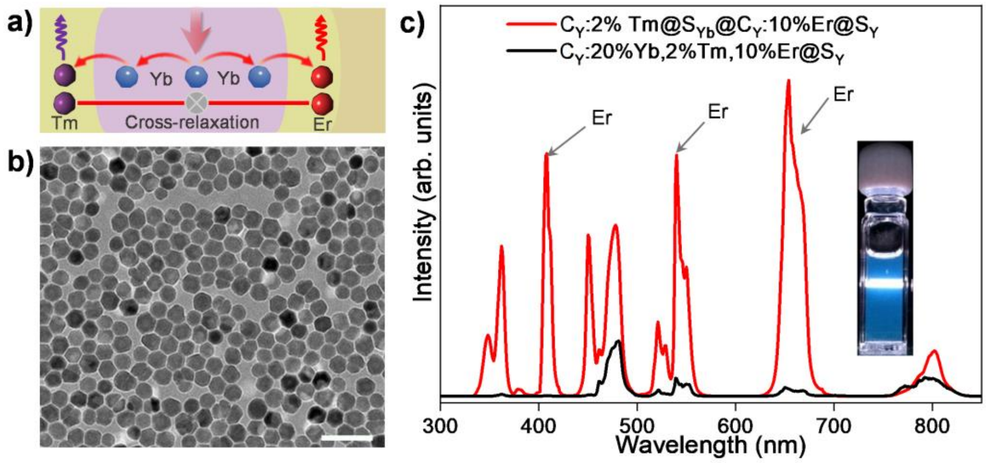

Supplementary Figure 31. Enhanced multiphoton emission from Tm^{3+} and Er^{3+} in dual activators doped core-shell-shell-shell nanoparticles. (a) Schematic representation of energy transfer processes in the α-$NaYF_4$:Tm@$NaYbF_4$@$NaYF_4$:Er @$NaYF_4$ nanoparticles. (b) TEM image of the α-$NaYF_4$:Tm@$NaYbF_4$@$NaYF_4$:Er @$NaYF_4$ nanoparticles. The scale bars is 100 nm. (c) Upconversion emission spectra for the α-$NaYF_4$:Tm@$NaYbF_4$@$NaYF_4$:Er@$NaYF_4$ and α-$NaYF_4$:Yb,Tm,Er@ $NaYF_4$ nanoparticles. The inset shows the luminescent photograph of the α-$NaYF_4$:Tm@$NaYbF_4$@$NaYF_4$:Er@$NaYF_4$ nanoparticles.

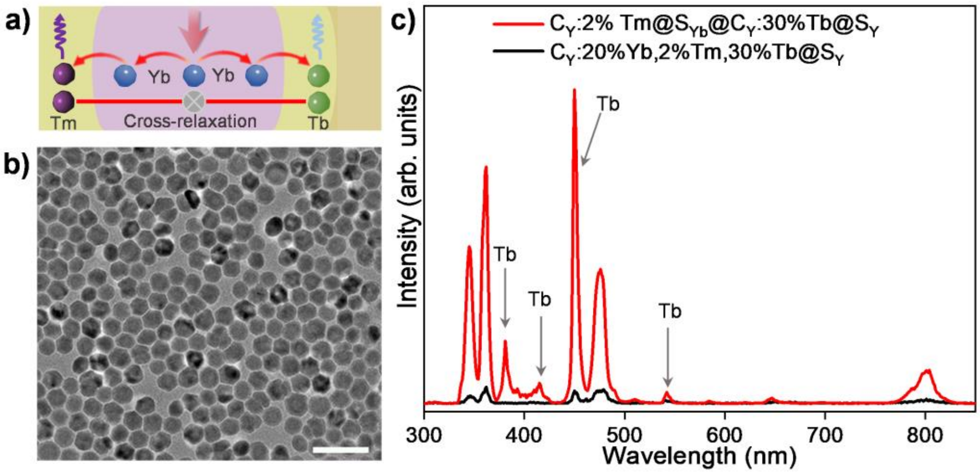

Supplementary Figure 32. Enhanced multiphoton emission from Tm^{3+} and Tb^{3+} in dual activators doped core-shell-shell-shell nanoparticles. (a) Schematic representation of energy transfer processes in the α-$NaYF_4$:Tm@$NaYbF_4$@$NaYF_4$:Tb @$NaYF_4$ nanoparticles. (b) TEM image of the α-$NaYF_4$:Tm@$NaYbF_4$@$NaYF_4$:Tb @$NaYF_4$ nanoparticles. The scale bars is 100 nm. (c) Upconversion emission spectra for the α-$NaYF_4$:Tm@$NaYbF_4$@$NaYF_4$:Tb@$NaYF_4$ and α-$NaYF_4$:Yb,Tm,Tb@ $NaYF_4$ nanoparticles.

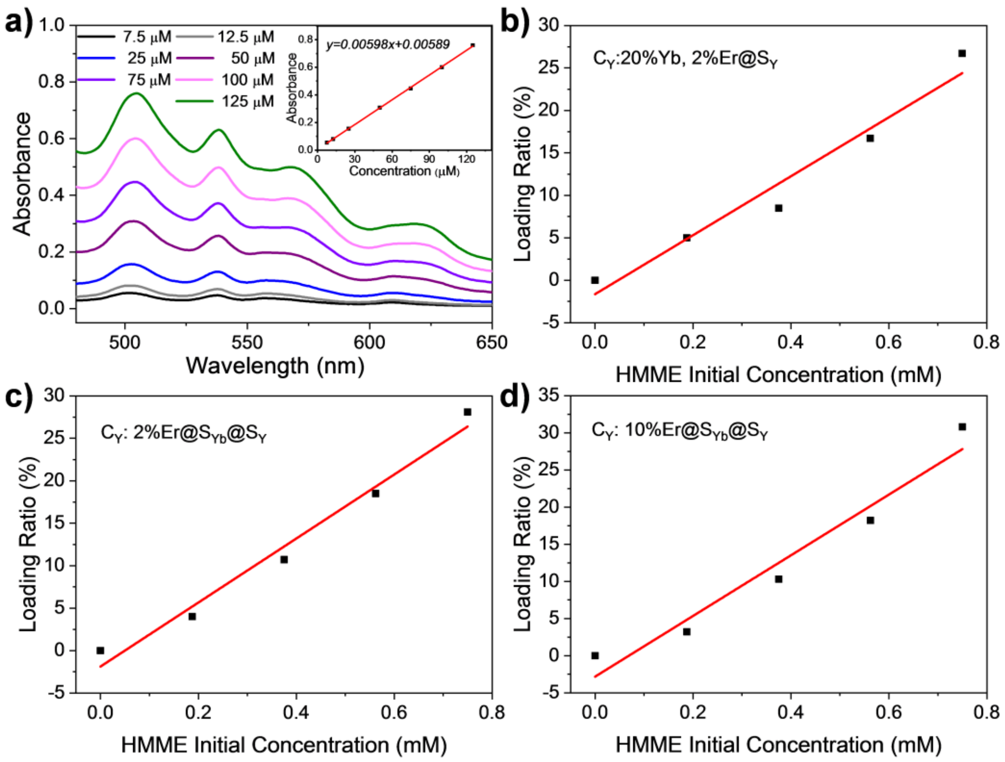

Supplementary Figure 33. Loading of HMME on various samples. (a) The absorbance curve of the HMME water solution at different concentrations and (inset) the relationship between HMME concentration and absorption at 504 nm. The absorption at 504 nm is much less affected by aggregation than the maximum absorption at 392 nm. HMME loading percentages of (b) α-$NaYF_4$:20%Yb, 2%Er@$NaYF_4$ (d_{SY} = 10.5±0.8 nm), (c) α-$NaYF_4$:2%Er@$NaYbF_4$@$NaYF_4$ (d_{SYb} = 8.3±0.7 nm, d_{SY} = 2.3±0.1 nm) (d) α-$NaYF_4$:10%Er@$NaYbF_4$@$NaYF_4$ (d_{SYb} = 8.3±0.7 nm, d_{SY} = 2.3±0.1 nm) Tween20-UCNPs with different concentration of HMME aqueous solution. The loading percentages determined by absorption spectra are nearly proportional to HMME initial concentration and no saturation can be observed. 25 wt % HMME (loading capacity of 0.41 mmol g^{-1}) was thus loaded on these UCNPs to investigate the $^1O_2$ production efficiency of the three different structures.

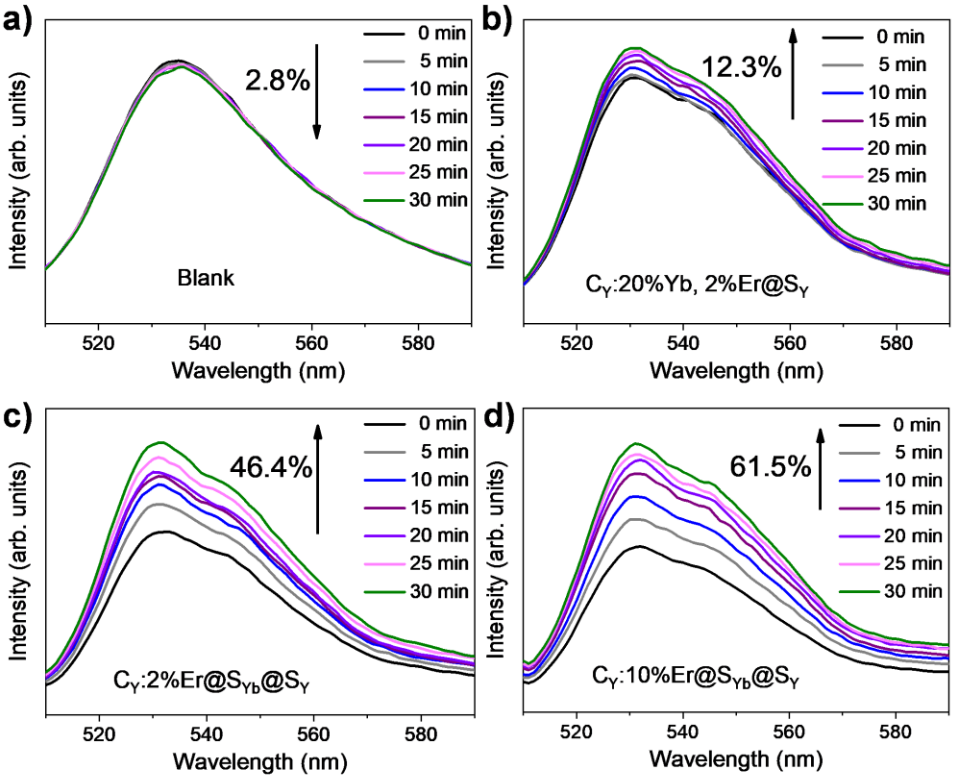

Supplementary Figure 34. Singlet oxygen production of HMME on various nanoparticles. Fluorescence spectra measured every 5 minutes of SOSG (10 μM) aqueous solution (a) and incubated with HMME-UCNPs in aqueous dispersion irradiated by 980 nm laser (24 W cm^{-2}): (b) α-$NaYF_4$:20%Yb, 2%Er@$NaYF_4$ (d_{SY} = 10.5±0.8 nm), (c) α-$NaYF_4$:2%Er@$NaYbF_4$@$NaYF_4$ (d_{SYb} = 8.3±0.7 nm, d_{SY} = 2.3±0.1 nm), (d) α-$NaYF_4$:10%Er@$NaYbF_4$@$NaYF_4$ (d_{SYb} = 8.3±0.7 nm, d_{SY} = 2.3±0.1 nm).

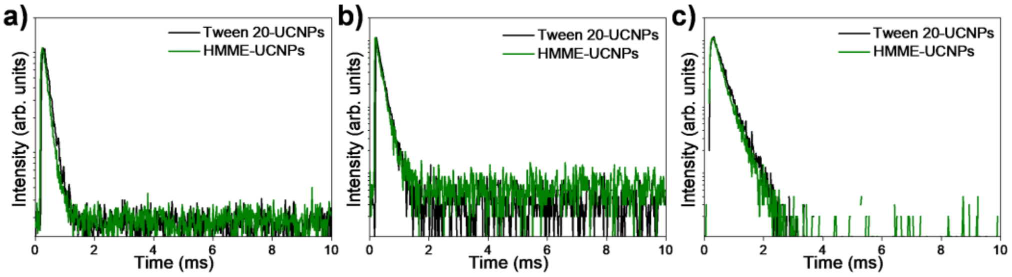

Supplementary Figure 35. Luminescence decays exclude Förster resonance energy transfer (FRET) mechanism. Luminescence decay curves for Tween 20 modified $NaYF_4$:10%Er@$NaYbF_4$@$NaYF_4$ nanoparticles (d_{SYb} = 8.3±0.7 nm, d_{SY} = 2.3±0.1 nm) before and after loading the photosensitizer, HMME. The decay curves were obtained by monitoring upconverted Er^{3+} emission at 407 nm (a), 540 nm (b) and 651 nm (c) under excitation of a pulsed laser at 980 nm, respectively. The invariable lifetimes exclude FRET mechanism.

Supplementary Table 1. Detailed dosage of precursors for synthesis of core-shell and core-shell-shell Nanoparticles.

Sample	Dosage of precursors	Size (nm)	Shell thickness (nm)
α-C_Y: x_1%Yb, y_1%Er	1 mmol NaTFA + 1-x_1-y_1 mmol YTFA + x_1 mmol YbTFA + y_1 mmol ErTFA	11.1±1.0	-
[1]α-C_Y: 20%Yb, 2%Er@S_Y	0.3 mmol $NaYF_4$:20%Yb,2%Er + 6.4 mmol NaTFA + 6.4 mmol YTFA	32.0±2.5	10.5±0.8
α-C_Y: 10%Er@S_{Yb}	0.3 mmol $NaYF_4$:10%Er + x_2 mmol NaTFA + x_2 mmol YbTFA	13.7±1.4 (x_2 = 0.18)<br>18.3±1.8 (x_2 = 1)<br>21.1±2.0 (x_2 = 1.57)<br>24.3±2.3 (x_2 = 2.56)<br>27.7±2.4 (x_2 = 4)<br>31.5±2.4 (x_2 = 4.6)<br>37.3±2.3 (x_2 = 5.5)	1.3±0.2<br>3.6±0.4<br>5.0±0.5<br>6.6±0.6<br>8.3±0.7<br>10.2±0.7<br>13.1±0.7
[2]α-C_Y: 10%Er@S_{Yb}@S_Y	0.3 mmol α-C_Y: 10%Er@S_{Yb} (d_{SYb} = 1.3±0.2) + 0.64 mmol NaTFA + 0.64 mmol YTFA	17.7±2.0	2.0±0.3
	0.3 mmol α-C_Y: 10%Er@S_{Yb} (d_{SYb} = 3.6±0.4) + 1.15 mmol NaTFA + 1.15 mmol YTFA	23.1±2.4	2.4±0.3
	0.3 mmol α-C_Y: 10%Er@S_{Yb} (d_{SYb} = 5.0±0.5) + 1.44 mmol NaTFA + 1.44 mmol YTFA	25.8±1.6	2.4±0.2
	0.3 mmol α-C_Y: 10%Er@S_{Yb} (d_{SYb} = 6.6±0.6) + 1.86 mmol NaTFA + 1.86 mmol YTFA	29.8±2.1	2.7±0.1
	0.3 mmol α-C_Y: 10%Er@S_{Yb} (d_{SYb} = 8.3±0.7) + y_2 mmol NaTFA + y_2 mmol YTFA[2]	30.3±2.0 (y_2 = 1.86)<br>32.2±2.2 (y_2 = 2.4)[2]<br>33.3±1.8 (y_2 = 3)<br>38.9±3.8 (y_2 = 7.4)	1.3±0.1<br>2.3±0.1<br>2.8±0.3<br>5.6±0.7
	0.3 mmol α-C_Y: 10%Er@S_{Yb} (d_{SYb} = 10.2±0.7) + 2.4 mmol NaTFA + 2.4 mmol YTFA	36.1±2.3	2.3±0.1
	0.3 mmol α-C_Y: 10%Er@S_{Yb} (d_{SYb} = 13.1±0.7) + 2.5 mmol NaTFA + 2.5 mmol YTFA	42.1±2.1	2.4±0.1
α-C_Y: 10%Er@S_Y@S_{Yb}@S_Y	0.3 mmol $NaYF_4$:10%Er + 0.2 (0.6) mmol NaTFA/YTFA → 4.2 (4.5) mmol NaTFA/YbTFA →2.6 (3) mmol NaTFA/YTFA	33.8±1.7<br>36.5±2.3	d_{SY1} = 1.1±0.1<br>d_{SY1} = 2.3±0.2

[1] Same dosage of different precursors was adopted for synthesis of β-C_Y: 20%Yb, 2%Er@S_Y, Li-C_Y: 20%Yb, 2%Er@Li-S_Y, α-C_Y: 20%Yb, x%Tm@S_Y, α-C_{Yb}: 30%Tb@S_Y, α-C_Y: 20%Yb, 2%Tm, 10%Er@S_Y, and α-C_Y: 20%Yb, 2%Tm, 30%Tb@S_Y NPs.

[2] Same dosage of different precursors was adopted for synthesis of β-C_Y: 10%Er@S_{Yb}@S_Y, Li-C_Y: 10%Er@Li-S_{Yb}@Li-S_Y, α-C_Y: x%Tm@S_{Yb}@S_Y, and α-C_Y: 30%Tb@S_{Yb}@S_Y NPs.

Supplementary Table 2**.** Fitting results of the decay curves at 407 nm (Supplementary Fig. 16a) for α-$NaYF_4$: Er (2-100mol%)@$NaYbF_4$@$NaYF_4$ core-shell-shell nanoparticles upon 980 nm irradiation.

**Sample**	**Monitoring State**	**τ_1 (ms)[1]**	**τ_1%**	**τ_2 (ms)**	**τ_2%**	**R^2**	**τ_{av} (ms)[2]**
**2%**	**Er^{3+}: $^2H_{9/2}\rightarrow{}^4I_{15/2}$**	**0.05**	**48.3**	**0.36**	**51.7**	**0.99**	**0.41**
**10%**				**0.77**	**100**	**0.99**	**0.77**
**30%**				**0.83**	**100**	**0.99**	**0.83**
**50%**		**0.18**	**76.1**	**0.26**	**23.9**	**0.99**	**0.44**
**70%**		**0.08**	**80.3**	**0.14**	**19.7**	**0.99**	**0.22**
**100%**		**0.07**	**100**			**0.99**	**0.07**

[1]Possibly arise from ion-ion interactions that produce nonradiative decay pathways.

[2]The average lifetime (τ_{av}) can be calculated using $\tau_{av} = \sum A_i \tau_i^2 / \sum A_i \tau_i$, where A_i is the pre-exponential for lifetime τ_i.

Supplementary Table 3. Upconversion quantum yields of different lanthanide-doped materials*.

**Sample**	**Size (nm)**	**QY (%)**	**Power density (W/cm^2)**	**Ref.**
α-$NaYF_4$:20%Yb,2%Er@$NaYF_4$	32.0±2.5	0.61±0.20	4.5	This work
α-$NaYF_4$:10%Er@$NaYbF_4$@$NaYF_4$	32.2±2.2	5.42±0.43	4.5	
α-$NaYF_4$:10%Er@$NaYbF_4$@$NaYF_4$ (d_{SY} = 5.6±0.7 nm)	38.8±3.8	6.34±0.48	4.5	
β-$NaYF_4$:10%Er@$NaYbF_4$@$NaYF_4$	26.1±4.3 × 37.2±3.9	5.08±0.39	4.5	
β-$NaYF_4$:10%Er@$NaYbF_4$@$NaYF_4$	31.9±1.3	6.82±0.50	4.5	
α-$NaYF_4$:2%Er@$NaYbF_4$@$NaYF_4$	~32	0.37±0.02	4.5	
α-$NaYF_4$:30%Er@$NaYbF_4$@$NaYF_4$	~32	2.59±0.11	4.5	
α-$NaYF_4$:50%Er@$NaYbF_4$@$NaYF_4$	~32	3.16±0.15	4.5	
α-$NaYF_4$:70%Er@$NaYbF_4$@$NaYF_4$	~32	2.01±0.13	4.5	
α-$NaErF_4$@$NaYbF_4$@$NaYF_4$	32.8±2.4	0.36±0.02	4.5	
α-$NaYF_4$:10%Er@$NaYF_4$@$NaYbF_4$@$NaYF_4$	33.8±1.7	3.00±0.32	4.5	
α-$NaYF_4$:10%Er@$NaYF_4$@$NaYbF_4$@$NaYF_4$	36.5±2.3	1.23±0.40	4.5	
$LiYF_4$:10%Er@$LiYbF_4$@$LiYF_4$	23.8±2.4 ×39.7±4.4	0.90±0.13	4.5	
$LiYF_4$:20%Yb, 2%Er@$LiYF_4$	23.1±2.8 ×38.4±4.5	0.23±0.02	4.5	
α-$NaYF_4$:8%Tm@$NaYbF_4$@$NaYF_4$	~32	0.48±0.04	4.5	
α-$NaYF_4$:20%Yb,0.5%Tm@$NaYF_4$**	~32	<0.1	4.5	
α-$NaYF_4$:30%Tb@$NaYbF_4$@$NaYF_4$**	~32	<0.1	4.5	
α-$NaYbF_4$:Tb@$NaYF_4$**	~32	<0.1	4.5	
β-$NaYF_4$:18%Yb,2%Er@$NaYF_4$	19.9±1.1	2.11±0.72	4.5	
β-$NaYF_4$:18%Yb,2%Er@$NaYF_4$	22	~2	4~5	8
β-$NaGdF_4$:22%Yb,2.5%Er@$NaYF_4$	20.1	0.89±0.05	50	11

β-$NaYF_4$:18%Yb,2%Er@$NaYF_4$	30	0.3	unknown	12
β-$NaYF_4$:18%Yb,2%Er@$NaYF_4$	38	1.83	unknown (2W)	13
β-$NaYF_4$: 33%Yb, 5%Er@$NaLuF_4$	49.8	4.0±0.5	63	14
β-$NaYF_4$:20%Yb,2%Er @$NaYF_4$	29.1±1	5.03±0.60	120	3
β-$NaYF_4$@$NaYbF_4$:8%Er@$NaYF_4$	29.4±1.2	5.42±0.45	120	3
β-$NaErF_4$@ $NaYF_4$***	19.7	0.17	unknown (2W)	13
β-$NaErF_4$@ $NaYF_4$***	38	5.2±0.3	10	15

*All α-$NaYF_4$:A@$NaYbF_4$@$NaYF_4$ UCNPs have similar structure with d_{SYb} ≈ 8.3 nm and d_{SY} ≈ 2.3 nm if not specified; all α-$NaYF_4$:Yb,A@$NaYF_4$ UCNPs have similar structure with d_{SY} ≈ 10.5 nm. There is a large variation in the d_{SYb} and d_{SY} of β-$NaYF_4$:Er@$NaYbF_4$@$NaYF_4$ UCNPs due to uncontrollable growth of β-$NaYbF_4$ shell in this work.

**Lower than the detection limit of our spectroscopy.

***UC process: Excited-state absorption.

Supplementary Table 4. Detailed dosage of precursors for synthesis of core-shell-shell-shell NPs.

Sample	Core	S1	S2	S3
[3]α-C_Y: 2%Tm@S_{Yb}@S_Y: 10%Er@S_Y	1 mmol NaTFA + 0.98 mmol YTFA + 0.02 mmol TmTFA	0.3 mmol C_Y:2%Tm + 4 mmol NaTFA + 4 mmol YbTFA	0.3mmol C_Y:2%Tm@S_{Yb} + 0.7 mmol NaTFA + 0.63mmol YTFA + 0.07mmol ErTFA	0.3 mmol C_Y: 2%Tm@S_{Yb}@S_Y: 10%Er + 2.4 mmol NaTFA + 2.4 mmol YTFA

[3]Same dosage of different precursors was adopted for synthesis of α-C_Y: 2%Tm@S_{Yb}@S_Y: 30%Tb@S_Y NPs.

Supplementary References

(1) Zhou, B. et al. Enhanced green upconversion luminescence in tetrahedral $LiYF_4$:Yb/Er nanoparticles by manganese(II)-doping: the key role of the host lattice. *Nanoscale* **10**, 2834-2840 (2018).

(2) Mai, H., Zhang, Y., Sun, L. & Yan, C. Highly efficient multicolor up-conversion emissions and their mechanisms of monodisperse $NaYF_4$:Yb,Er core and core/shell-structured nanocrystals. *J. Phys. Chem. C* **111**, 13721-13729 (2007).

(3) Liu, Q. et al. Single upconversion nanoparticle imaging at sub-10 W cm^{-2} irradiance. *Nat. Photon.* **12**, 548-553 (2018).

(4) Zhao, J. et al. Single-nanocrystal sensitivity achieved by enhanced upconversion luminescence. *Nat. Nanotechnol.* **8**, 729-734 (2013).

(5) Wang, L. et al. Reversible near-infrared light directed reflection in a self-organized helical superstructure loaded with upconversion nanoparticles. *J. Am. Chem. Soc.* **136**, 4480−4483 (2014).

(6) Chen, X. et al. Confining energy migration in upconversion nanoparticles towards deep ultraviolet lasing. *Nat. Commun.* **7**, 10304 (2016).

(7) Bian, W. et al. Direct identification of surface defects and their influence on the optical characteristics of upconversion nanoparticles. *ACS Nano* 12, 3623-3628 (2018).

(8) Homann C. et al. $NaYF_4$:Yb,Er/$NaYF_4$ core/shell nanocrystals with high upconversion luminescence quantum yield. *Angew. Chem. Int. Ed.* 57, 8765-8769 (2018).

(9) Tian, B. et al. Low irradiance multiphoton imaging with alloyed lanthanide nanocrystals. *Nat. Commun.* **9**, 3082 (2018).

(10) Rabie, H. et al. NIR biosensing of neurotransmitters in stem cell-derived neural interface using advanced core–shell upconversion nanoparticles. *Adv. Mater.* **31**, 1806991 (2019).

(11) Li, X. et al. Engineering homogeneous doping in single nanoparticle to enhance upconversion efficiency. *Nano Lett.* **14**, 3634-3639 (2014).

(12) Zhou, B. et al. Photon upconversion through Tb^{3+} - mediated interfacial energy transfer. *Adv. Mater.* **27**, 6208-6212 (2015).

(13) Chen, Q. et al. Confining excitation energy in Er^{3+} - sensitized upconversion nanocrystals through Tm^{3+} - mediated transient energy trapping. *Angew. Chem. Int. Ed.* **56**, 7713-7717 (2017).

(14) Fischer, S. et al. Precise Tuning of surface quenching for luminescence

enhancement in core-shell lanthanide-doped nanocrystals *Nano Lett.* **16**, 7241-7247 (2016).

(15) Johnson, N. J. J. et al. Direct evidence for coupled surface and concentration quenching dynamics in lanthanide-doped nanocrystals. *J. Am. Chem. Soc.* **139**, 3275-3282 (2017).
